# A combined GWAS approach reveals key loci for socially-affected traits in Yorkshire pigs

**DOI:** 10.1038/s42003-021-02416-3

**Published:** 2021-07-20

**Authors:** Pingxian Wu, Kai Wang, Jie Zhou, Dejuan Chen, Anan Jiang, Yanzhi Jiang, Li Zhu, Xiaotian Qiu, Xuewei Li, Guoqing Tang

**Affiliations:** 1 Farm Animal Genetic Resources Exploration and Innovation Key Laboratory of Sichuan Province, Sichuan Agricultural University, Chengdu, Sichuan China; 2 College of Life Science, Sichuan Agricultural University, Yaan, Sichuan China; 3 National Animal Husbandry Service, Beijing, Beijing, China

**Keywords:** Heritable quantitative trait, Genetic association study, Genetics

## Abstract

Socially affected traits in pigs are controlled by direct genetic effects and social genetic effects, which can make elucidation of their genetic architecture challenging. We evaluated the genetic basis of direct genetic effects and social genetic effects by combining single-locus and haplotype-based GWAS on imputed whole-genome sequences. Nineteen SNPs and 25 haplotype loci are identified for direct genetic effects on four traits: average daily feed intake, average daily gain, days to 100 kg and time in feeder per day. Nineteen SNPs and 11 haplotype loci are identified for social genetic effects on average daily feed intake, average daily gain, days to 100 kg and feeding speed. Two significant SNPs from single-locus GWAS (SSC6:18,635,874 and SSC6:18,635,895) are shared by a significant haplotype locus with haplotype alleles ‘GGG’ for both direct genetic effects and social genetic effects in average daily feed intake. A candidate gene, *MT3*, which is involved in growth, nervous, and immune processes, is identified. We demonstrate the genetic differences between direct genetic effects and social genetic effects and provide an anchor for investigating the genetic architecture underlying direct genetic effects and social genetic effects on socially affected traits in pigs.

## Abstract (web summary)

Pingxian Wu and Kai Wang et al. perform single-locus and haplotype-based GWAS in Yorkshire pigs to evaluate genetic factors involved in socially affected traits, like feed intake and weight gain. These results indicate several genetic factors that may ultimately be targeted to improve livestock breeding.

## Introduction

Social interactions inevitably occur among groups of individuals and these interactions influence the genetic variation of a trait in livestock^1^. In current pig production systems, pigs are penned together into contemporary groups, social interactions, and observed phenotypes genetically contribute to socially affected traits of individuals and their group mates^2^. Socially affected traits are influenced both by the genes of the individual itself (direct genetic effects, DGE) and by the genotype of other individuals in the same group (social genetic effects, SGE, also called indirect genetic effects)^2–5^. Previous research has proven that many phenotypes are affected by both DGE and SGE and the contribution of SGE to total genetic variance is large in pigs, such as growth rate, body weight (BW)^6,7^, feed intake, backfat thickness (BFT), muscle depth^2^, behavior^8,9^, and average daily gain (ADG)^10,11^. The existence of SGE affect the estimation of DGE and therefore result in unfavorable consequence on genetic improvement of complex traits. Until recently, the genetic architecture of socially affected traits that involve both DGE and SGE is still poorly understood in pigs.

With the availability of genomic information, GWAS have increased the understanding of the contributions of genetic variants toward various complex traits. However, previous studies have mainly focused on DGE and have not considered the contributions of SGE. For many phenotypes, DGE and SGE can be directly quantified using a social genetic model^2^. Based on the social genetic model, the classical (direct) genetic model is expanded with SGE^12^. This model simultaneously contains both DGE and SGE that enables researchers to simultaneously estimate DGE and SGE values.

Detecting the genetic architecture of DGE and SGE may provide deeper insight into a better understanding of complex traits. To date, only a few studies have identified some SNPs associated with SGE. Using the Illumina 60 K SNP BeadChip, an important SNP was identified to be associated with DGE and SGE in chickens^13^. In pigs, five SNPs on chromosome 6 associated with social ADG were detected with an Illumina panel^14^. Additionally, in our previous study, a total of 27 SNPs associated with six growth traits were identified using whole-genome sequences (WGS) of 40 Yorkshire pigs (Zhejiang Tianpeng Group Co., Ltd. Zhejiang, China)^15^. However, because of the limited sample size, the study was not efficient in detecting credible associations with DGE and SGE.

Assessing the impact of SGE on socially affected traits is challenging. Haplotype-based GWAS can capture those genetic variants that are not detected by single-locus GWAS^16^. Therefore, to better investigate the genetic architecture of socially affected traits in pigs, the present study used both GWAS approaches to estimate the DGE and SGE on each trait by employing a social genetic model, WGS of 60 pigs, and genotyping of 1204 Yorkshire pigs with Illumina 50 K SNP chips. Therefore, the main objectives of this study were (i) to construct haplotype loci based on imputed WGS data and identify associations between haplotype-DGE and haplotype-SGE by GWAS; (ii) to perform single-locus analysis to explore SNPs associated with socially affected traits by considering both DGE and SGE in Yorkshire pigs; (iii) to compare the results of GWAS based on haplotypes and SNPs for DGE and SGE; and (iv) to combine single-locus and haplotype-based GWAS to reveal important SNPs and genes for DGE and SGE in pigs.

## Results

### Phenotype statistics

Summary statistics for all phenotypic data from 1204 Yorkshire pigs were shown in Table 1. Based on the social genetic model, the estimated accuracies and deregressed EBVs of DGE and SGE for each trait were presented in Table 2. All the DGE and SGE values were used for further analysis.

**Table 1:** Descriptive statistics of phenotypes for eight socially–affected traits in Yorkshire pigs.

Trait	Unit	Number	Mean ± SD	Max	Min
ADFI	kg	1112	1.87 ± 0.34	2.84	0.53
ADG	kg	1112	0.74 ± 0.14	1.26	0.15
B100	mm	1026	9.27 ± 2.34	19.98	4.23
D100	day	1112	183.61 ± 22.37	391.93	100
FCR	kg/kg	1112	2.53 ± 0.31	3.74	1.51
RFI	g	1112	7.89 ± 216.07	905.05	−923.5
TPD	min/day	1107	64.51 ± 15.91	123.24	28.09
FS	g/min	1107	30.79 ± 8.01	57.76	13.51

*ADFI* average daily feed intake, *ADG* average daily gain, *B100* backfat thickness to 100 kg, *D100* days to 100 kg, *FCR* feed conversion ratio, *RFI* residual feed intake, *TPD* time in feeder per day, *FS* feeding speed, *Number* number of phenotypic records, *Mean* arithmetic mean, *SD* standard deviation, *Max* maximum, *Min* minimum.

**Table 2:** Summary of the estimated accuracies and deregressed EBVs for direct genetic (DGE) and effects social genetic effects (SGE) in Yorkshire pigs.

Trait		Number	Estimated accuracies (Mean ± SD)	Deregressed EBVs (Mean ± SD)
DGE	ADFI	1112	0.80 ± 0.17	1.77E-02 ± 0.22
	ADG	1112	0.80 ± 0.17	5.40E-04 ± 0.18
	B100	1026	0.40 ± 0.22	6.00E-02 ± 1.38
	D100	1112	0.55 ± 0.22	7.85E-02 ± 19.49
	FCR	1112	0.30 ± 0.21	7.64E-03 ± 0.24
	RFI	1112	0.44 ± 0.24	14.43 ± 194.37
	TPD	1107	0.55 ± 0.22	−6.09E-03 ± 8.07
	FS	1107	0.44 ± 0.23	0.11 ± 3.87
SGE	ADFI	1112	0.68 ± 0.19	2.99E-05 ± 0.07
	ADG	1112	0.60 ± 0.21	3.95E-05 ± 0.07
	B100	1026	0.40 ± 0.22	−1.37E-03 ± 0.62
	D100	1112	0.23 ± 0.18	−8.67E-03 ± 7.99
	FCR	1112	0.30 ± 0.21	8.16E-05 ± 0.16
	RFI	1112	0.12 ± 0.14	0.08 ± 119.57
	TPD	1107	0.51 ± 0.22	0.01 ± 2.55
	FS	1107	0.44 ± 0.23	−1.02E-03 ± 1.63

*ADFI* average daily feed intake, *ADG* average daily gain, *B100* backfat thickness to 100 kg, *D100* days to 100 kg, *FCR* feed conversion ratio, *RFI* residual feed intake, *TPD* time in feeder per day, *FS* feeding speed, *Number* number of phenotypic records, *Mean* arithmetic mean, *SD* standard deviation.

### Evaluation of sequencing data and genotype imputation

A total of 60 pigs were sequenced and the statistic results were list in Supplementary Data 1. After initial quality filtering, 3.0-TB clean data with the average mapped read depth of 17.11 was retained. The mean reads were 336,176,275 for each sample. After mapping to the pig reference genome, the mean mapped reads and the mean uniquely mapped reads were 312,374,099 and 293,904,451 for each sample, respectively. The average mapping and unmapping ratios were 92.92 and 1.28% per individual, respectively.

**Fig. 1:**
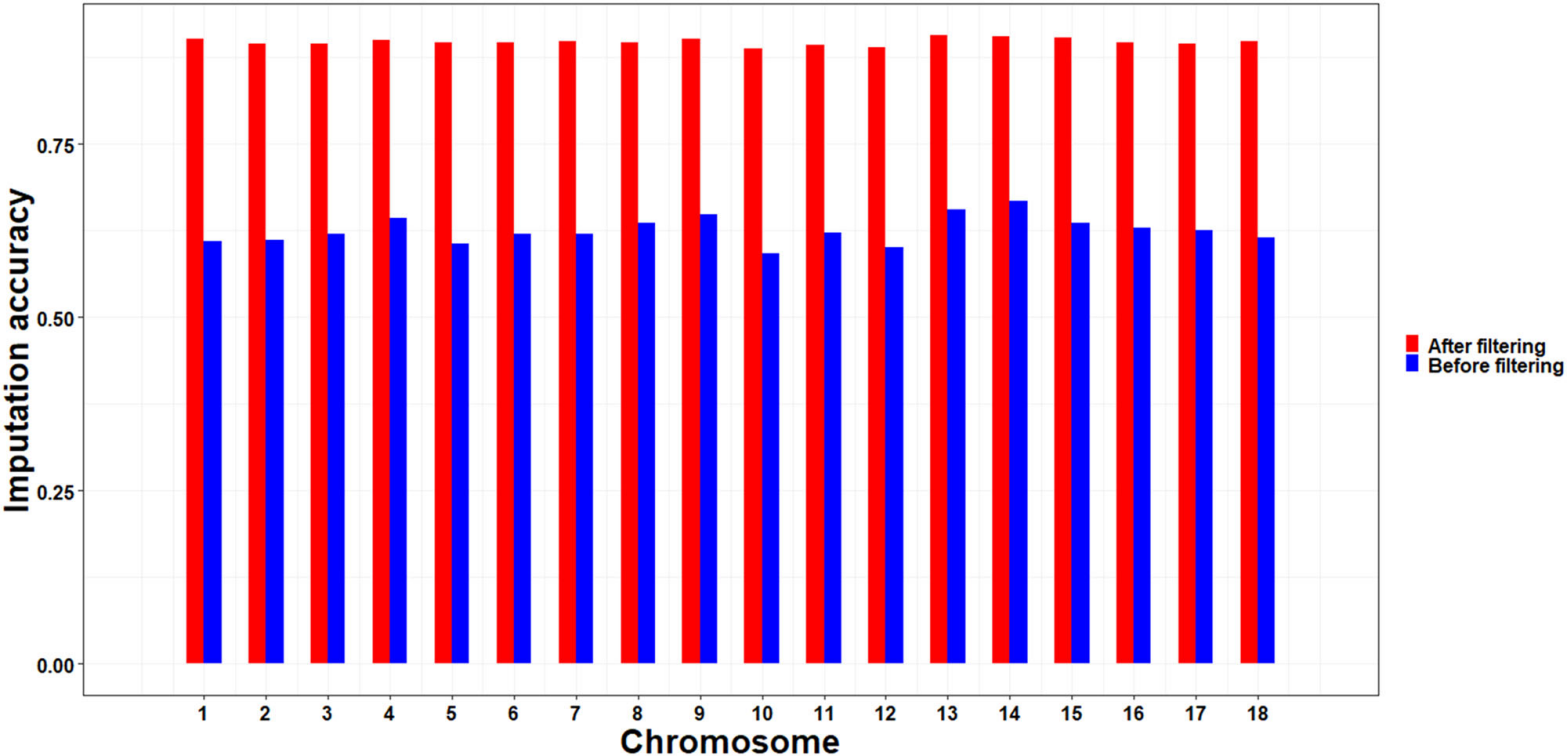
Imputation accuracy for each chromosome in Yorkshire pigs. Accuracy of imputation based on Beagle *R*^2^ before and after filtering (Beagle *R*^2^ < 0.8). “Before filtering” for the blue part, “After filtering” for the red part.

This study conducted genotype imputation from a 50 K SNP chip to WGS data in Yorkshire pigs using a two-breed reference population (20 Landrace and 40 Yorkshire pigs). In total, 36,969 and 14,539,997 SNPs were identified in the target and reference populations, respectively. Beagle 5.1 software was used to impute the WGS reference panel across the 1204 genotyped pigs. After genotype imputation, a total of 14,539,997 SNPs with an average accuracy of 0.63 were available for this study. For each chromosome, the average imputation accuracy was in the range of 0.59–0.67 (Fig. 1). The SSC14 had the highest imputation accuracy (0.67), while SSC10 had the lowest imputation accuracy (0.59). After quality control, a total of 3,072,572 SNPs with an average imputation accuracy of 0.90 were retained for further analyses.

**Fig. 2:**
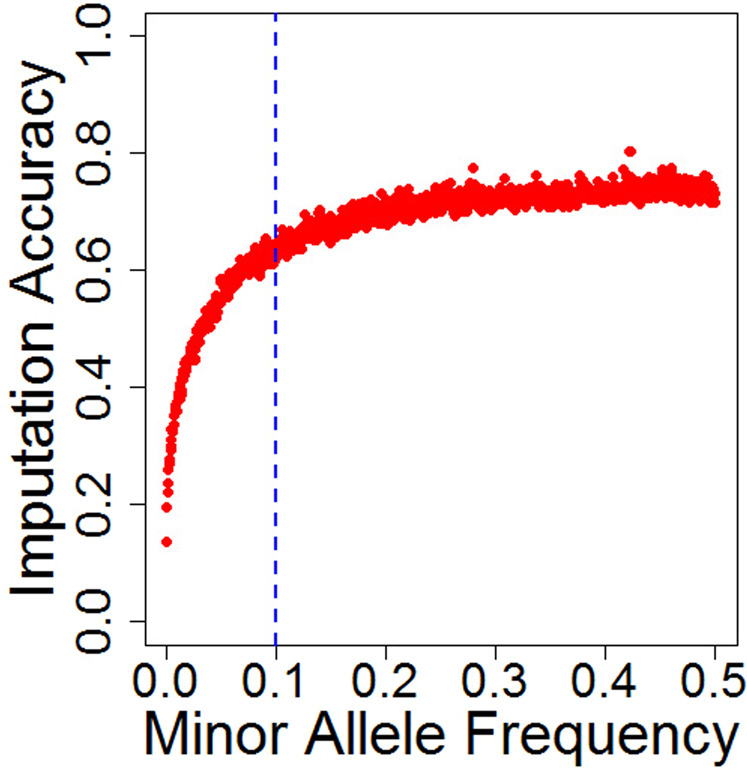
Imputation accuracy against minor allele frequency (MAF). SNPs were divided into bins of SNPs with common MAF.

The average imputation accuracies of imputed SNPs with different minor allele frequency (MAF) were calculated and are shown in Fig. 2. The imputation accuracies were comparatively stable for different MAFs where the MAF was higher than 0.1. However, the imputation accuracy decreased sharply when the MAF was lower than 0.1.

### Single-locus GWAS for eight socially affected traits

#### For average daily feed intake (ADFI)

**Fig. 3:**
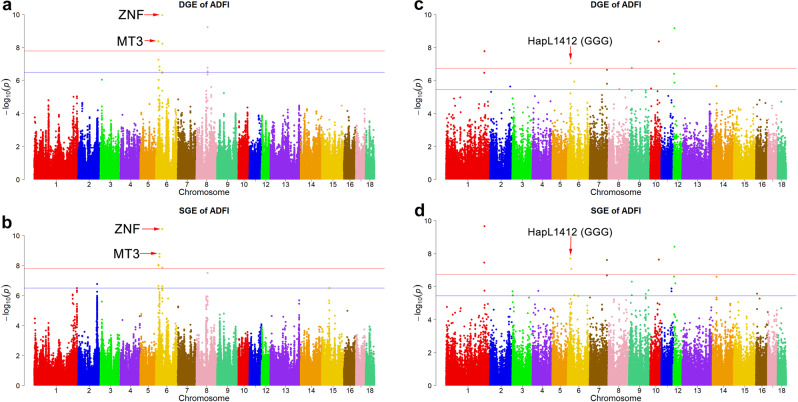
GWAS results for direct genetic effects (DGE) and social genetic effects (SGE) in average daily feed intake (ADFI). **a** for DGE using imputed-GWAS; **b** for SGE using imputed-GWAS; **c** for DGE using haplotype-based GWAS; **d** for SGE using haplotype-based GWAS. For imputed-GWAS, the horizontal red and blue lines indicate the genome-wide (1.63 × 10^−8^) and suggestive (3.25 × 10^−7^) level, respectively. For haplotype-based GWAS, the horizontal red and blue lines indicate the genome-wide (1.82 × 10^−7^) and suggestive (3.64 × 10^−6^) level, respectively.

The imputed-GWAS results for DGE in ADFI are shown in Table 3, Supplementary Data 2, and Fig. 3a. The imputed-GWAS identified 413 SNPs ($P \; < \; {3.25\times 10}^{-7}$) associated with DGE at the suggestive threshold. Of these 413 SNPs, the 17 SNPs ($P \; < \; {1.63\times 10}^{-8}$) located on SSC6 and SSC8 showed a significant signal. The most significant SNP ($P={1.07\times 10}^{-10}$) was located on SSC6: 46,448,592. In a narrow 9.75 kb region (18.64–18.65 Mb) on chromosome 6, there were ten consecutive genome-wide significant SNPs that showed significant peaks. In the region of SSC8: 79.51–79.53 Mb, five genome-wide significant SNPs showed high signals. A λ of less than 1.05 is considered to indicate a lack of population stratification^17^. The λ calculated for the DGE on ADFI was 1.01; implying no population stratification (Supplementary Fig. 1a). For chip-based GWAS, the significant threshold was set at $P={2.70\times 10}^{-5}$. On the basis of the 50-K chip data, a total of 97 SNPs were identified (Supplementary Fig. 2). Three SNPs from the chip-based GWAS were also in the imputed GWAS ($P \; < \; {2.70\times 10}^{-5}$, Supplementary Table 1).

**Table 3:** Single-locus GWAS for direct genetic effects (DGE) in Yorkshire pigs.

Trait	SSC	Position (bp)	Range (Mb)	Allele	*P*_value	Candidate Gene
DGE_ADFI	6	18640605	18.14–19.14	A/G	4.09E–09	*CDH1*, *CDH3*, *ZFP90*, *BBS2*, *MT4*, *MT3*, *MT1A*, *NUP93*, *MIR138-2*, *HERPUD1*, *SLC12A3*, *NLRC5*, *CPNE2*, *FAM192A*
	6	18635874	18.14–19.14	C/G	4.40E–09	
	6	18635895	18.14–19.14	A/G	4.40E–09	
	6	18639708	18.14–19.14	C/T	4.40E–09	
	6	18641480	18.14–19.14	C/T	4.40E–09	
	6	18642431	18.14–19.14	C/G	4.40E–09	
	6	18643649	18.14–19.14	A/G	4.40E–09	
	6	18644213	18.14–19.14	T/C	4.40E–09	
	6	18645373	18.15–19.15	A/T	4.40E–09	
	6	18645626	18.15–19.15	A/G	4.40E–09	
	6	46451435	45.95–46.95	A/G	5.89E–09	*ZNF566*, *ZFP82*, *ZFP14*, *ZNF793*, *ZNF527*, *ZNF569*, *U2*, *ZNF570*, *ZFP30*, *WDR87*
	6	46448592	45.95–46.95	T/G	1.07E–10	
	8	79506792	79.01–80.01	C/T	5.98E–10	*IQCM*
	8	79525267	79.03–80.03	A/T	5.98E–10	
	8	79529184	79.03–80.03	G/A	5.98E–10	
	8	79529190	79.03–80.03	T/C	5.98E–10	
	8	79529891	79.03–80.03	C/T	5.98E–10	
DGE_ADG	6	25649764	25.15–26.15	C/T	2.21E–09	*CDH11*
	6	25651261	25.15–26.15	T/C	2.43E–09	
	8	79506792	79.01–80.01	C/T	1.85E–09	*IQCM*
	8	79525267	79.03–80.03	A/T	1.85E–09	
	8	79529184	79.03–80.03	G/A	1.85E–09	
	8	79529190	79.03–80.03	T/C	1.85E–09	
	8	79529891	79.03–80.03	C/T	1.85E–09	

*DGE_ADFI* direct genetic effects of average daily feed intake, *DGE_ADG* direct genetic effects of average daily gain, *SSC* chromosome, *Range* range of significant chromosome region.

For SGE on ADFI, a total of 26 SNPs reached the suggestive threshold using imputed GWAS (Table 4, Supplementary Data 2, and Fig. 3b). Among these, 14 genome-wide significant SNPs were detected on chromosome 6, which had the highest signals (Fig. 3b). The λ was equal to 1.00, which indicates no population stratification (Supplementary Fig. 1b). A total of 89 SNPs were found from the chip-based GWAS (Supplementary Fig. 2). Among them, two overlapped with the imputed GWAS ($P \; < \; {2.70\times 10}^{-5}$, Supplementary Table 1).

**Table 4:** Single-locus GWAS for social genetic effects (SGE) in Yorkshire pigs.

Trait	SSC	Position (bp)	Range (Mb)	Allele	*P*_value	Candidate Gene
SGE_ADFI	6	18635874	18.14–19.14	C/G	8.94E–09	*CDH1*, *CDH3*, *ZFP90*, *BBS2*, *MT4*, *MT3*, *MT1A*, *NUP93*, *MIR138-2*, *HERPUD1*, *SLC12A3*, *NLRC5*, *CPNE2*, *FAM192A*
	6	18635895	18.14–19.14	A/G	8.94E–09	
	6	18639708	18.14–19.14	C/T	8.94E–09	
	6	18641480	18.14–19.14	C/T	8.94E–09	
	6	18642431	18.14–19.14	C/G	8.94E–09	
	6	18643649	18.14–19.14	A/G	8.94E–09	
	6	18644213	18.14–19.14	T/C	8.94E–09	
	6	18645373	18.15–19.15	A/T	8.94E–09	
	6	18645626	18.15–19.15	A/G	8.94E–09	
	6	18640605	18.14–19.14	A/G	1.00E–08	
	6	25649764	25.15–26.15	C/T	1.67E–09	*CDH11*
	6	25651261	25.15–26.15	T/C	2.68E–09	
	6	46451435	45.95–46.95	A/G	1.39E–08	*OVOL3*, *POLR2I*, *TBCB*, *CAPNS1*, *COX7A1*, *ZNF146*, *ZNF565*, *ZNF567*, *ZNF461*, *ZNF382*, *ZNF260*, *ZNF566*, *ZFP82*, *ZFP14*, *ZNF793*, *ZNF527*, *ZNF569*, *U2*, *ZNF570*, *ZFP30*, *WDR87*
	6	46448592	45.95–46.95	T/G	3.63E–11	
	8	79506792	79.01–80.01	C/T	3.16E–08	*IQCM*
	8	79525267	79.03–80.03	A/T	3.16E–08	
	8	79529184	79.03–80.03	G/A	3.16E–08	
	8	79529190	79.03–80.03	T/C	3.16E–08	
	8	79529891	79.03–80.03	C/T	3.16E–08	
SGE_ADG	6	25649764	25.15–26.15	C/T	3.00E–09	*CDH11*
	6	25651261	25.15–26.15	T/C	3.68E–09	
	8	79506792	79.01–80.01	C/T	3.17E–09	*IQCM*
	8	79525267	79.03–80.03	A/T	3.17E–09	
	8	79529184	79.03–80.03	G/A	3.17E–09	
	8	79529190	79.03–80.03	T/C	3.17E–09	
	8	79529891	79.03–80.03	C/T	3.17E–09	

*SGE_ADFI* social genetic effects of average daily feed intake, *SGE_ADG* social genetic effects of average daily gain, *SSC* chromosome, *Range* range of significant chromosome region.

**Fig. 4:**
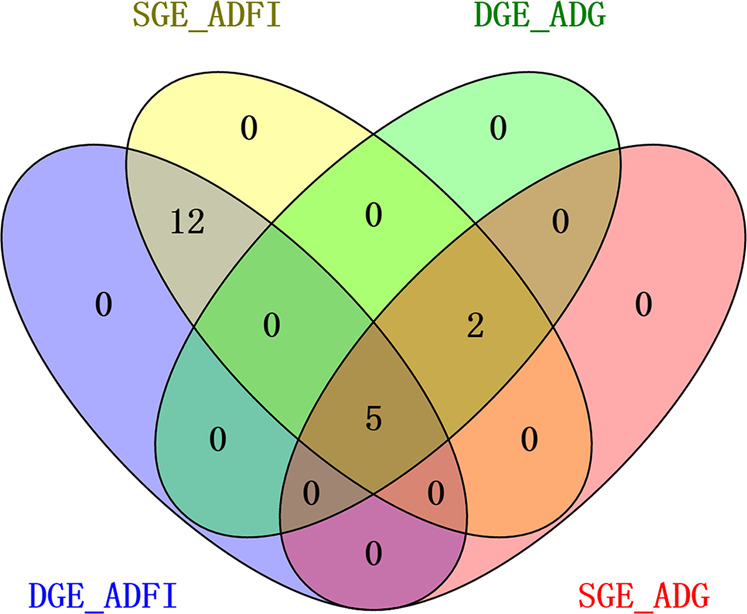
The common SNPs were shared by both direct genetic effects (DGE) and social genetic effects (SGE). Venn diagram of common significant SNPs of DGE and SGE in average daily feed intake (ADFI) and average daily gain (ADG).

A comparison between the imputed GWAS of DGE and SGE on ADFI revealed a total of 17 common SNPs shared between DGE and SGE (Fig. 4). Notably, the use of SGE identified associations for ADFI: two SNPs (SSC6: 25,649,764, $P={1.67\times 10}^{-9}$ and SSC6: 25,651,261, $P={2.68\times 10}^{-9}$) were significant for SGE but were not significant for DGE ($P \; > \; {1.00\times 10}^{-7}$).

#### For ADG

**Fig. 5:**
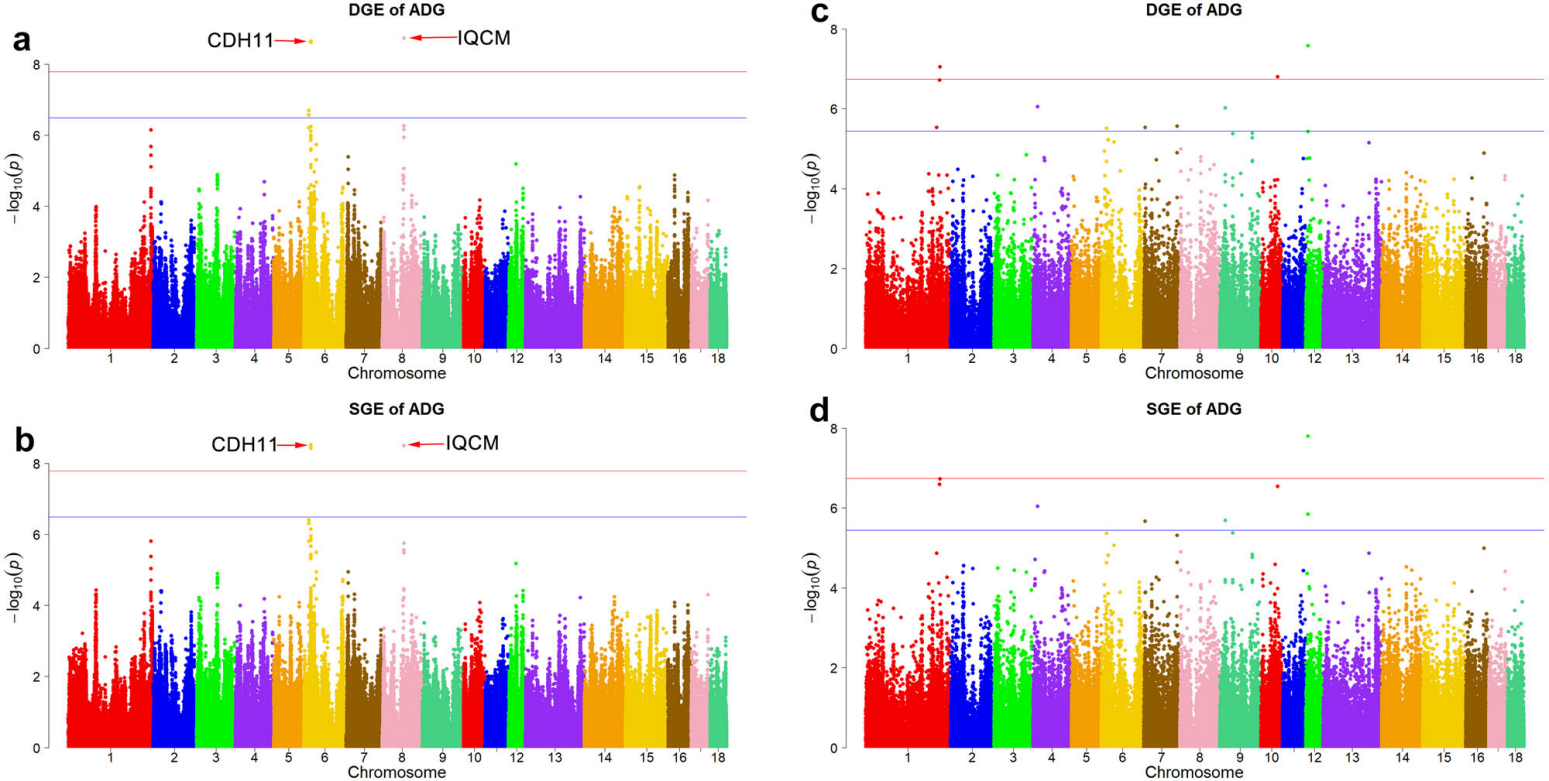
GWAS results for direct genetic effects (DGE) and social genetic effects (SGE) in average daily gain (ADG). **a** for DGE using imputed-GWAS; **b** for SGE using imputed-GWAS; **c** for DGE using haplotype-based GWAS; **d** for SGE using haplotype-based GWAS. For imputed-GWAS, the horizontal red and blue lines indicate the genome-wide (1.63 × 10^−8^) and suggestive (3.25 × 10^−7^) level, respectively. For haplotype-based GWAS, the horizontal red and blue lines indicate the genome-wide (1.82 × 10^−7^) and suggestive (3.64 × 10^−6^) level, respectively.

On the basis of imputed GWAS, seven common SNPs reached the genome-wide significant threshold ($P={1.63\times 10}^{-8}$) and were identified for both DGE (Table 3 and Figs. 4,  5a) and SGE in ADG (Table 4 and Figs. 4,  5b). Of these SNPs, five consecutive loci located within SSC8: 79.51–79.53 Mb were identified with a *P* value of ${1.85\times 10}^{-9}$ for DGE and ${3.17\times 10}^{-9}$ for SGE. Ten consecutive SNPs were detected at a suggestive threshold for DGE in ADG in the region between 18.64 and 18.65 MB on chromosome 6 (Supplementary Data 2). The λ was 1.02 for DGE (Supplementary Fig. 1c) and 1.03 for SGE (Supplementary Fig. 1d). These λ values were similar to the values found for DGE and SGE in ADFI. Moreover, in the chip-based GWAS, 87 SNPs were identified for DGE and 85 SNPs were identified for SGE ($P \; < \; {2.70\times 10}^{-5}$, Supplementary Fig. 2).

#### For the other six traits

Using imputed GWAS, no significant SNPs were identified for either DGE or SGE for the backfat thickness to 100 kg (B100), days to 100 kg (D100), feed conversion ratio (FCR), residual feed intake (RFI), time in feeder per day (TPD), and feeding speed (FS) traits (Supplementary Fig. 3). However, two peaks were found for D100; one of which was located on SSC6 for DGE and the other on SSC5 for SGE. For RFI, a distinct peak located on SSC10 was found for both DGE and SGE.

In the chip-based GWAS, the total number of SNPs detected ($P \; < \; {2.70\times 10}^{-5}$) was 4 for B100, 15 for D100, 20 for RFI, 3 for FS, and 5 for TPD. For SGE, the total number of SNPs detected ($P \; < \; {2.70\times 10}^{-5}$) was 1 for B100, 5 for RFI, 1 for FS, and 1 for TPD (Supplementary Fig. 2). Among them, three SNPs were also detected by imputed GWAS for the B100, D100, and FS traits ($P \; < \; {2.70\times 10}^{-5}$, Supplementary Table 1).

### Haplotype-based GWAS for eight socially affected traits

#### For ADFI

Four significant haplotype loci were identified for DGE (Supplementary Table 2 and Fig. 3c) and seven for SGE (Supplementary Table 3 and Fig. 3d). These were distributed on SSC1, SSC6, SSC7, SSC10, and SSC12 ($P \; < \; {1.82\times 10}^{-7}$). Among these, four common haplotype loci (HapL1834, HapL910, HapL1412, and HapL1193) were shared by DGE and SGE. Only the *MT3* gene was located within the haplotype HapL1412 “GGG”, with a haplotype frequency of 0.05. Furthermore, 12 suggestive haplotype loci were detected for DGE and 17 for SGE (${1.82\times 10}^{-7} \; < \; P \; < \; {3.64\times 10}^{-6}$, Supplementary Table 4). The λ was 1.06 for DGE (Supplementary Fig. 4a) and 1.08 for SGE (Supplementary Fig. 4b); implying no population stratification.

#### For ADG

Three haplotype loci surpassing the significance threshold ($P={1.82\times 10}^{-7}$) were detected for DGE (Supplementary Table 2 and Fig. 5c) and one was detected for SGE (Supplementary Table 3 and Fig. 5d). The haplotype loci (HapL910) were shared by DGE and SGE. In addition, this haplotype was shared by ADFI and ADG with a haplotype frequency of 0.03. A total of ten suggestive haplotype loci were identified for DGE and SGE (Supplementary Table 4). The λ was 1.04 for DGE (Supplementary Fig. 4c) and 1.03 for SGE (Supplementary Fig. 4d); implying no population stratification.

#### For D100 and B100

**Fig. 6:**
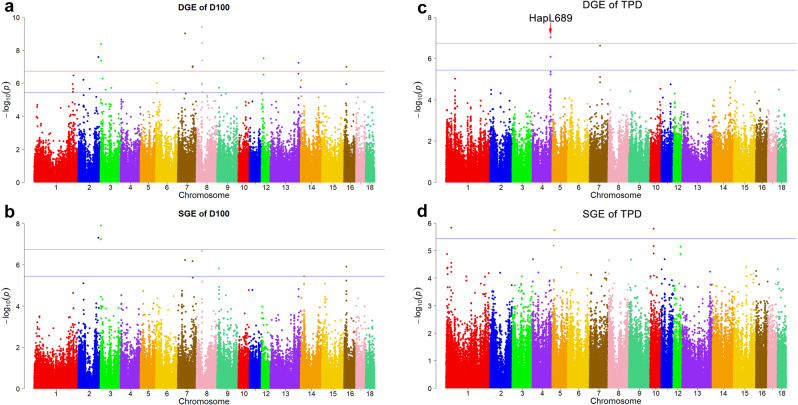
Haplotype-based GWAS results for direct genetic effects (DGE) and social genetic effects (SGE). **a** for DGE in days to 100 kg (D100); **b** for SGE in D100; **c** for DGE of the time in feeder per day (TPD); **d** for SGE of TPD. The horizontal red and blue lines indicate the genome-wide (1.82 × 10^−7^) and suggestive (3.64 × 10^−6^) level, respectively.

When considering the DGE and SGE, we identified 20 different haplotype loci that reached the significance threshold ($P={1.82\times 10}^{-7}$) for D100 (Supplementary Tables 2, 3 and Figs. 6a, b). Among these, three common haplotype loci (HapL36 HapL1601, and HapL213) were detected for DGE and SGE. Eighteen candidate genes were located within these significant regions. The *SDK1* gene within the significant haplotype HapL36 was shared by DGE and SGE. On basis of the suggestive level ($P={3.64\times 10}^{-6}$), a total of 21 haplotype loci were detected for DGE and 13 for SGE (Supplementary Table 4). For B100, no haplotype loci were found to be associated with DGE (Supplementary Fig. 5a) or SGE (Supplementary Fig. 5b).

#### For FCR and RFI

No significant haplotype loci were detected for DGE or SGE (Supplementary Fig. 6); however, five suggestive haplotype loci (${1.82\times 10}^{-7} \; < \; P \; < \; {3.64\times 10}^{-6}$) were identified for DGE (three for FCR and two for RFI) and one for SGE on RFI (Supplementary Table 4 and Supplementary Fig. 6).

#### For TPD and FS

Significant haplotype loci located on SSC4: 130,340,342–130,390,531 were identified for the DGE on TPD (Fig. 6c) and significant haplotype loci were detected for the SGE on FS (Supplementary Fig. 7). Furthermore, 15 different haplotype loci that surpassed the suggestive level ($P={3.64\times 10}^{-6}$) were identified for DGE and SGE (Supplementary Table 4).

### Comparing single-locus and haplotype-based GWAS

Haplotype-based GWAS identified more associations than single-locus GWAS for the DGE and SGE on the eight socially affected traits in pigs. Importantly, the significant HapL1412 with haplotype allele “GGG” overlapped with two significant SNPs (SSC6: 18,635,874 and SSC6: 18,635,895) for both DGE and SGE on ADFI. A candidate gene, *MT3*, was found in these regions. Furthermore, multiple significant SNPs and haplotype loci were shared by different traits, which indicates the pleiotropism.

## Discussion

The socially affected traits are influenced by multiple genes. This study is designed to investigate DGE and SGE for eight socially affected traits using imputed WGS, as there is strong evidence that these traits are socially affected in pigs^2,8–10,18^. Using the social genetic model^12^, we estimated the DGE and SGE of each socially affected trait in Yorkshire pigs. Then the single-locus and haplotype-based GWAS were performed to investigate associations for DGE and SGE using imputed data.

Different imputation strategies can result in different levels of accuracy^19^. The use of multi-breed reference populations is an effective way to improve the accuracy of imputation^19,20^. However, a large genetic distance between the reference and target populations can result in extremely low accuracies (less than 0.49)^21^. Landrace and Yorkshire pigs are bred according to the same breeding goals and are therefore likely genetically similar^22^. Additionally, because of the small effective population size for pigs^23^, the imputation parameter of effective population size was set at 100 in the Beagle 5.1 software. After imputation, the average accuracy at the whole-genome level was 0.63, and 3,072,572 SNPs had a Beagle *R*^2^ higher than 0.80 in the imputed WGS dataset. The imputation accuracy observed in the current study was higher than that reported in other studies^19–21^, for example, a study using a ten-breed reference population of 168 pigs and using imputation from low- or high-density SNP data to WGS resulted in relatively low accuracy (0.39–0.49)^21^. Our results were more similar to the accuracy obtained from a multi-breed reference population of cattle (>0.70)^19,20^. Thus, the present study indicates that using mixed reference populations with similar genetic backgrounds can result in reasonable imputation accuracy in pigs. The imputation accuracy increased with reference population size^24^. In this study, a total of 40 sequenced pigs was used. Although the reference population size is smaller than that used in previous studies (>100) for pigs^25^, this study also obtained a high imputation accuracy, and therefore, the imputed data can provide important information regarding socially affected traits in pigs.

Imputation accuracy is heavily affected by the MAF^26^. Rare variants with a low MAF result in poor imputation accuracy and therefore decrease the average imputation accuracy. In our study, the accuracy decreased sharply when the MAF was lower than 0.10. Filtering out the SNPs with MAF < 0.10 from the imputed data increased the average imputation accuracy from 0.63 to 0.71. In our study, the imputation accuracy increased with the MAF, which is in agreement with previous studies in livestock^21,24,27,28^. Rare variants are expected to have larger effects on complex traits than common variants^29^. It is also harder to construct the haplotype background using rare SNPs and they decrease the set of template haplotypes for genotype imputation because they are observed only a few times in reference genotype datasets^26^. Thus, genotype imputation of rare SNPs does not provide high accuracy levels. It is important to investigate imputation methods for rare variants in further studies.

In the processes of natural selection and the artificial selection of pigs, complex traits have developed high levels of genetic variation. The single-locus analysis focuses on identifying SNPs and mining functional genes for complex traits^13^, but this method has only identified a small fraction of genetic variants in pigs. Haplotype-based GWAS is a complementary method that intensifies the benefits from linkage disequilibrium (LD) and enables the evaluation of the genetic determinants of complex traits. The use of haplotype information in GWAS would likely be beneficial for detecting further genetic variants^30^. However, haplotype-based GWAS using imputed WGS has not yet been reported in pigs. The rare variants may contribute large effects to complex traits^29^. Our analysis were done on imputed sequence data, which may contain imputation errors^31^. To alleviate this issue, further quality control was also conducted on the imputed sequence genotypes, rare variants with a MAF lower than 0.01 were moved. After quality control, the average MAF is 0.30 for chip-based data, 0.27 for imputed data, and 0.09 for haplotype alleles. Our results show that haplotype-based GWAS can be effective and can provide more genetic information than single-locus GWAS. These results are in agreement with previous studies; for example, haplotype-based GWAS in plants^32^ and cattle^16^ captured genetic variants that were not identified by single-locus analysis. Importantly, by combining single-locus and haplotype-based GWAS, we confirmed the presence of two important SNPs (SSC6: 18,635,874 and SSC6: 18,635,895) for DGE and SGE in ADFI. Therefore, our results highlight the advantages of haplotype-based GWAS for detecting genetic variants that influence complex traits in pigs. The power of GWAS is limited by sample size and SNP density. Using the imputed WGS instead of the 50 K SNP chip is powerful to investigate socially affected traits in pigs. Although only 1204 imputed WGS were used in this study, our results also provided important genetic variation for socially affected traits. Further studies are recommended to validate these results using a larger sample size.

Socially affected traits are known to be affected by SGE, but the mechanisms of SGE are not fully known. Many studies have been conducted to understand the genetic architecture of complex traits and they have identified many important SNPs and genes associated with these traits^33^. However, these studies have mainly focused on investigations of DGE, despite the majority of complex traits in pigs being affected by social interactions among individuals^2^. Pigs are highly social animals, so ignoring SGE could incorrectly estimate genetic variance and restrict genetic improvement.

Recently, numerous studies have shown that using social genetic models during genetic evaluation can enhance the genetic improvement of socially affected traits in pigs^1,10^. However, few studies have investigated the genetic architecture of DGE and SGE in pigs^14,15,34^. On the basis of our GWAS results, we found that the significant loci and candidate genes of DGE are different from SGE for each trait in Yorkshire pigs. These results are in agreement with previous studies, for example, a distinct difference was found between DGE and SGE for socially affected traits in laying hens^13^, and a study based on single-step GWAS found only one QTL (SSC6: 19.9–20.9 Mb) for DGE and SGE on ADG in pigs^34^. The population composition may affect the estimation of DGE and SGE. An experimental design using groups comprising two families is optimal for estimating SGE^35^. Our group’s composed of numerous families because all pigs in our study were from a commercial pig performance testing station. The group design in this study may have affected the estimation of DGE and SGE; however, it is difficult to conduct an experiment containing only two families from a commercial breeding program. Moreover, in our study, all pigs were derived from the same herd and there were 20 pigs in each group, which made it easier to estimate DGE and SGE^5^.

Our results provide further evidence for the genetic differences in DGE and SGE with regard to socially affected traits. Economically important traits are selected by artificial selection when based on the classical model that only considers DGE. SGE is, therefore, less frequently selected when compared with DGE. The complex traits in pigs are highly polygenic, and more research is required to understand the genetic architecture of the SGE on the complex traits of pigs.

To assess whether the identified SNPs associated with socially affected traits in our study replicate previously identified QTL, we compared our results with the PigQTL database based on the genome position of each SNP and QTL. A total of 78 important QTL were found to overlap with previously identified QTL for DGE of complex traits in pigs (Supplementary Table 5). No previous QTL has been reported to be associated with SGE. SGE has been evaluated to play an important role in livestock^12^. Thus, the investigation of the genetic architecture of SGE has important implications for pig breeding programs.

In our study, a total of 56 candidate genes associated with SGE and DGE were detected. Gene enrichment analysis showed that enriched pathways were associated with nucleic acid, calcium, and metal ion binding (Supplementary Table 6). In particular, two candidate genes, *IQCM* and *CDH11*, were associated with both DGE and SGE on the ADG and ADFI traits. *CDH11* gene encodes a calcium-dependent glycoprotein and mediates calcium-dependent cell-cell adhesion^36^. The *CDH11* gene is essential for tissue development, regulation of cell proliferation, and survival^37,38^. This gene has also been associated with postnatal bone development in mice^39^. Previous studies have shown that the *CDH11* gene is associated with growth traits in cattle including weaning weight^40^ and RFI^41^. In pigs, this gene was found to be associated with fat and meat quality traits^42^ and to regulate neural development and cell motility^43^. In this important chromosome region, six QTL for growth traits were overlapped, including ADG^44^ and BW^45^ in pigs. Additionally, these significant SNPs were located on the QTL for health traits^46^.

In the region of 45.95–46.95 Mb on SSC6, numerous zinc-finger protein family genes were found to be associated with both DGE and SGE. The zinc-finger protein is one of the most abundant classes of transcription factors and regulates cell growth and differentiation. According to our gene ontology (GO) analysis, these genes regulate transcription, nucleic acid binding, and metal ion binding. Furthermore, this chromosome region overlapped with four reported QTL affecting ADG^44,47,48^ and eating behavior^49^ traits in pigs. Thus, daily interactions (SGE) between group pigs would influence their health. Interestingly, the significant SNPs within the region SSC6: 45.95–46.95 Mb were located within 20 QTL known to be associated with health traits^46,50^ in pigs (https://www.animalgenome.org/).

In the region of SSC6: 18.14–19.14 Mb, *MT3* gene was found in both single-locus and haplotype-based GWAS for socially affected traits. This gene encodes a growth inhibitory factor that regulates many biological processes, particularly within the nervous and immune systems, and thereby influences health. *MT3* plays an important role in zinc homeostasis and cell death^51^, and inhibits cell growth under zinc-deficient conditions^52^. Interestingly, our study also identified numerous zinc-finger protein family genes to be associated with DGE and SGE in SSC6: 45.95–46.95 Mb.

The previous studies identified several QTL on SSC6 for DGE and SGE in pigs^14,15,34^. The QTL (SSC6: 18.14–19.14 Mb) in the current study was close to the reported QTL (SSC6: 19.9–20.9 Mb) for both DGE and SGE on ADG in pigs^34^. Furthermore, there were eight reported QTL associated with growth traits in the region of SSC6: 18.14–19.14 Mb. Among them, five QTL were related to ADG^53^ and feeding intake^54^. The significant SNPs associated with DGE and SGE are located in nine reported QTL for health traits^46^. And, it was reported that three QTL located on SSC6: 18.14–19.14 Mb in pigs were associated with social interaction traits, and health traits^49,55^.

In summary, we report the study employing combined single-locus and haplotype-based GWAS to identify the genetic architecture of socially affected traits that are influenced by both DGE and SGE in pigs. Our study shows the feasibility of mapping genomic variants that underlie SGE and provides genomic information for socially affected traits in pigs. These results provide evidence that the genomic architecture of SGE and DGE is different for socially affected traits. Hence, we recommend the use of both DGE and SGE to evaluate genetic architecture for socially affected traits in pigs.

## Methods

### Ethics declarations

All experimental procedures were performed in accordance with the Institutional Review Board (IRB14044) and the Institutional Animal Care and Use Committee of the Sichuan Agricultural University under permit number DKY-B20140302.

### Animals and housing

During the period of 2017–2019, phenotypic data were collected for Yorkshire pigs from the national nucleus pig breeding farm of New Hope Group, Co., Ltd. (Sichuan, China) using the Osborne FIRE Pig Performance Testing System (Osborne, KS, United States). As target population, a total of 1204 Yorkshire pigs were placed in a temperature-controlled room at 25 ± 2 °C and relative humidity of 65–80% during the period of performance test from 30 to 110 kg. As reference population, a total of 60 pigs (20 Landrace and 40 Yorkshire pigs) were randomly selected from core populations. These pigs were group-housed in cement-floor pens (20 pigs in each pen) and the nutrient requirements were met as recommended by the National Research Council (NRC 2012).

### Phenotypic data

A total of 1112 pigs’ phenotypic data were collected, including ADG (kg/d), D100, B100, ADFI (kg/d), RFI, FCR, TPD (min/d), and FS (g/min). In this study, each pig was labeled with a unique electric identification tag on ear that was detected by the Osborne FIRE Pig Performance Testing System. The feed time, feed consumption, and BW were recorded at each visit to the feeder for each pig. At the end of the performance test, BFT between the third and fourth last ribs of each pig was calculated by PIGLOG 105B ultrasound machine (SFK Technology, Søborg, Denmark). ADG was calculated as linear regressions of BW from 30 to 110 kg on the performance test days. ADFI was calculated based on the total amount of recorded total feed intake (TFI) divided by the number of corresponding feed days at the feeder. TPD was calculated based on the total amount of recorded total time divided by the number of corresponding feed days (min/d), and FS = ADFI/TPD (g/min)^56,57^. FCR, D100, B100, and RFI were calculated as follows^58^:

1 $${{{{\mathrm{FCR}}}}}=\frac{{{{{\mathrm{TFI}}}}}}{{{{{\rm{Weight}}}}}_{2}-{{{{\rm{Weight}}}}}_{1}}$$

2 $${{{\rm{D}}}}100={{{\rm{tested}}}}\,{{{\rm{days}}}}+(100-{{{{\rm{Weight}}}}}_{2})\;\times\; \frac{{{{\rm{tested}}}}\,{{{\rm{days}}}}-{{{\rm{A}}}}}{{{{{\rm{Weight}}}}}_{2}}$$

3 $${{{\rm{B}}}}100={{{\rm{BFT}}}}+\left(100-{{{{\rm{Weight}}}}}_{2}\right)\times \frac{{{{\rm{BFT}}}}}{{{{{\rm{Weight}}}}}_{2}-B}$$

4 $${{{\rm{RFI}}}}={{{\rm{ADFI}}}}-14.1{{{\rm{ADG}}}}-2.83{{{\rm{BFT}}}}-110.9{{{\rm{AMW}}}}$$

5 $${{{\rm{AMW}}}}=\frac{\left({{{{\rm{Weight}}}}}_{2}^{1.6}-{{{{\rm{Weight}}}}}_{1}^{1.6}\right)}{1.6\times \left({{{{\rm{Weight}}}}}_{2}-{{{{\rm{Weight}}}}}_{1}\right)}$$

where ${{{{\bf{Weight}}}}}_{{{{\bf{1}}}}}$ and ${{{{\bf{Weight}}}}}_{{{{\bf{2}}}}}$ are weights at the start and end of the performance test, respectively; ${{{\bf{tested}}}}$ ${{{\bf{days}}}}$ is the duration of the performance test in days. ${{{\bf{A}}}}$ is 50.775 for males and 46.415 for females; ${{{\bf{B}}}}$ is −7.277 for males and −9.440 for females. A and B were calculated based on an actual dataset of performance tests involving 5000 pigs^59^. The true days and B100 were firstly obtained based on two data that were the closest to 100 kg using linear interpolation. Then the nonlinear models of D100 and B100 were constructed based on linear interpolation as models (2) and (3). Finally, the A and B were calculated based on models (2) and (3) using the NLIN procedure in SAS software, respectively. BFT is the tested backfat thickness at the end of a performance test. AMW is an average metabolic BW.

### The deregressed EBVs of DGE and SGE

The DGE and SGE were estimated for eight socially affected traits using a social genetic effect model^12^, as follows:

6 $${{{\boldsymbol{y}}}}={{{\boldsymbol{Xb}}}}+{{{{\boldsymbol{Z}}}}}_{{{{\boldsymbol{D}}}}}{{{{\boldsymbol{a}}}}}_{{{{\boldsymbol{D}}}}}+{{{{\boldsymbol{Z}}}}}_{{{{\boldsymbol{S}}}}}{{{{\boldsymbol{a}}}}}_{{{{\boldsymbol{S}}}}}+{{{\boldsymbol{Wl}}}}+{{{\boldsymbol{V}}}}{{{\boldsymbol{g}}}}+{{{\boldsymbol{e}}}}$$

where ${{{\boldsymbol{y}}}}$ is the vector of phenotypic values; ${{{\boldsymbol{b}}}}$ is the vector of fixed effects, including sex, test year and month, birth year and month effects; ${{{{\boldsymbol{a}}}}}_{{{{\boldsymbol{D}}}}}$ and ${{{{\boldsymbol{a}}}}}_{{{{\boldsymbol{S}}}}}$ are vectors of DGE and SGE, respectively; ${{{\boldsymbol{l}}}}$ is the vector of random litter effects; ${{{\boldsymbol{g}}}}$ is the vector of random group effects in which the pigs were penned during the performance test; $e$ is the random residual vector; ${{{\bf{X}}}}$, ${{{{\boldsymbol{Z}}}}}_{{{{\boldsymbol{D}}}}}$, ${{{{\boldsymbol{Z}}}}}_{{{{\boldsymbol{S}}}}}$, ${{{\boldsymbol{W}}}}$,and ${{{\boldsymbol{V}}}}$ are the incidence matrices of ${{{\boldsymbol{b}}}}$, ${{{{\boldsymbol{a}}}}}_{{{{\boldsymbol{D}}}}}$, ${{{{\boldsymbol{a}}}}}_{{{{\boldsymbol{S}}}}}$, ${{{\boldsymbol{l}}}}$, and ${{{\boldsymbol{g}}}}$, respectively. This model was implemented by AI-REML in DMU software^60^. The estimated accuracies of DGE and SGE were calculated based on the formula $r=\sqrt{1-{s}_{e}^{2}/{\sigma }_{a}^{2}}$, ${s}_{e}^{2}$ is the error variance of DGE and SGE, ${\sigma }_{a}^{2}$ is the corresponding genetic variance. Based on the estimated accuracies of DGE and SGE, their deregressed breeding values (EBVs) were obtained based on the formula $D{{{\rm{eregressed}}}}$ ${EBVs}={g}_{i}/{r}_{i}^{2}$, ${g}_{i}$ is the EBVs of $i$th individual, ${r}_{i}^{2}$ is the square of estimated accuracies for $i$th individual^61^. Then, the deregressed EBVs were used to implement for association analysis.

### Genomic DNA extraction

The ear tissue samples were collected and stored in 75% alcohol. Genomic DNA from 1264 ear tissues was extracted using the Tissues Genomic DNA (Omega Bio-Tek, Norcross, GA, USA) kit according to the manufacturer’s instructions, and then the quality and quantity were measured using a Nanodrop-2000 spectrophotometer. The genomic DNA with the ratio of light absorption (A260/280) between 1.8 and 2.0, concentration ≥50 ng/µL, and total volume ≤50 µL were eligible.

### Genotyping by Illumina Porcine SNP50K BeadChip

A total of 1204 Yorkshire pigs, 442 boars and 762 gilts, were genotyped by the Illumina Porcine 50K SNP Chip (Neogen, Lincoln, NE, USA), which contained 50,697 SNPs. Firstly, the SNPs with no position information and located on sex chromosomes were removed from the genotype data, which contains 11,874 SNPs. Then, quality control of the genotype data was performed using PLINK software^62^. The SNPs with call rate < 0.90, MAF < 0.05, and Hardy–Weinberg equilibrium test (HWE) <10^−6^, were excluded from the dataset. After quality control, a total of 1854 SNPs were removed. Finally, a total of 36,969 SNPs were used as target genotype data.

### Whole-genome sequencing and SNP calling

The WGS reference data were obtained for 60 pigs (20 Landrace and 40 Yorkshire pigs), with 150 bp paired-end reads on the Illumina HiSeq PE150 platform. The sequencing was performed by BGI Co., Ltd. (Wuhan, China). After sequencing, the quality of raw reads was checked with a Phred score of 20 as the minimum to filter the adapter polluted reads and multiple *N* reads (where *N* > 10% of one read) to produce clean reads by FastQC (http://www.bioinformatics.bbsrc.ac.uk/projects/fastqc/). Then the clean reads were mapped to the pig reference genome (Sscrofa11.1) using BWA (version 0.7.15) software with the parameters mem -t 10 -k 32 -M^63^. The SAM files produced from BWA were converted to BAM files using SAMtools (version 1.19)^64^. The potential PCR duplicates were removed by MarkerDuplicates utility in Picard release 1.119 (https://sourceforge.net/projects/picard/files/picardtools/1.119/). After that, BAM files were used to call SNPs using GATK (version 3.5) software^65^ with multi-sample approaches. The raw SNPs generated from GATK were filtered with QualByDepth (QD) < 2.0, FisherStrand (FS) < 60.0, RMSMappingQuality (MQ) < 40.0, MappingQualityRankSumTest < –12.5, and ReadPosRankSumTest < –8.0. After the initial filtering, a total of 21,104,245 SNPs remained. For further analyses, the SNPs with MAF >0.05, missing rate <0.1, HWE <10^−6^, read depth (dp) >6, and the SNPs located on autosomes were considered as reference genotype data.

### Imputation from 50 K chip to WGS

Genotype imputation between target and reference genotype data were performed by Beagle (version 5.1)^66^ with default parameter settings, except for setting the effective population size to 100^22^. The imputation accuracy of each SNP was assessed using the Beagle *R*^2^, which is the estimated squared correlation between the estimated allele dosage and the true allele dosage^67^. A two-breed reference population (20 Landrace and 40 Yorkshire pigs) with a small genetic distance^22^ was used to perform genotype imputation from 50 K SNPs to WGS. After imputation, to maintain a balance between the average accuracy and the number of SNPs, SNPs with a Beagle *R*^2^ < 0.8 were excluded. Furthermore, SNPs with MAF less than 0.01 and HWE less than 10^−6^ were removed from imputed data. Finally, a total of 3,072,572 SNPs were retained for further analyses.

### Constructing haplotype loci

Haplotype loci were identified from the imputed WGS data in 1204 pigs. Haplotype loci were detected for each chromosome as proposed by Gabriel et al.^68^ using PLINK v1.90 software^62^. The parameters were set to “-blocks no-pheno-req -blocks-max-kb 1000 -blocks-strong-lowci 0.8 -geno 0.1”. A haplotype block containing two or more SNPs with high LD was defined, and loci with a confidence interval of *r*^2^ higher than 0.8 were considered into one block. Then, the haplotype calling and identification of haplotype alleles were performed by GHap package^69^. Furthermore, haplotype loci were transformed into multi-allelic markers, and the haplotype genotype matrix was used to perform GWAS. Using the imputed data with 3,072,572 SNPs, a total of 33,708 haplotype loci were constructed and each haplotype loci contained 34.8 alleles. Finally, 274,741 haplotype alleles with MAF >0.01 were retained.

### Association analysis

GWAS were performed independently for eight socially affected traits by considering both DGE and SGE using GEMMA software^65^. Before association analysis, the centered genotypes were used to estimate the *n* × *n* genomic relationship matrix between the individuals. The genomic relatedness matrix was calculated as follows:

7 $$G=\frac{1}{p}\mathop{\sum }\limits_{i=1}^{p}{\left({X}_{i}-{1}_{n}{\bar{x}}_{i}\right)\left({X}_{i}-{1}_{n}{\bar{x}}_{i}\right)}^{T}$$

where $G$ is the genomic relatedness matrix between the individuals; $n$ is the number of individuals; $p$ is the number of genotypes; $i$ represents the $i$th SNP; X denote *n* × *p* matrix of genotypes; ${X}_{i}$ as its $i$th column representing genotypes of $i$th SNP; ${\bar{x}}_{i}$ as the sample mean of $i$th SNP; ${1}_{n}$ as a *n* × 1 vector of 1’s.

The deregressed EBVs were used to perform single-locus GWAS (including chip-based GWAS and imputed-GWAS) and haplotype-based GWAS, the following unified mixed linear model was used:

8 $${{{\bf{y}}}}={{{\bf{Xm}}}}+{{{\bf{Wa}}}}+{{{\bf{e}}}}$$

$${{{\boldsymbol{a}}}} \sim {{{{{\mathbf{MVN}}}}}}\left({{{\bf{0}}}},{{{\boldsymbol{G}}}}{{{{\boldsymbol{\sigma }}}}}_{{{{\boldsymbol{a}}}}}^{{{{\bf{2}}}}}\right)$$

$${{{\boldsymbol{e}}}} \sim {{{{{\mathbf{MVN}}}}}}\left({{{\bf{0}}}},{{{\boldsymbol{I}}}}{{{{\boldsymbol{\sigma }}}}}_{{{{\boldsymbol{e}}}}}^{{{{\bf{2}}}}}\right)$$

where ${{{\bf{y}}}}$ is the vector of the deregressed EBVs of DGE or SGE; ${{{\bf{m}}}}$ is the SNP effects; ${{{\bf{a}}}}$ is the vector of residual polygenic effects; ${{{\bf{e}}}}$ is the vector of random residuals; ${{{\bf{X}}}}$ and ${{{\bf{W}}}}$ are the incidence matrices for ${{{\bf{m}}}}$ and ${{{\bf{a}}}}$, respectively; *G* is a genomic relationship matrix, ${{{\boldsymbol{I}}}}$ is an identity matrix; MVN denotes multivariate normal distribution.

The genome-wide significant threshold value was determined using the Bonferroni correction method^70^. For chip-based GWAS, the genome-wide significant and suggestive levels were set as *P* = 0.05/*N*_1_ = 1.35 × 10^−6^ and *P* = 1/*N*_1_ = 2.70 × 10^−5^, respectively, where *N*_1_ is the number of analyzed SNPs. For imputed-GWAS, the genome-wide significant and suggestive levels were set as *P* = 0.05/*N*_2_ = 1.63 × 10^−8^ and *P* = 1/*N*_2_ = 3.25 × 10^−7^, respectively, where *N*_2_ is the number of analyzed SNPs. For haplotype-based GWAS, the genome-wide significant and suggestive levels were calculated as *P* = 0.05/*N*_3_ = 1.82 × 10^−7^ and *P* = 1/*N*_3_ = 3.64 × 10^−6^, respectively, where *N*_3_ is the number of studied haplotype alleles.

The Manhattan plots were drawn using qqman package^71^. Genomic inflation factor (λ) was calculated to judge the extent of false-positive signals with estlambda function in GenABEL package (${{{\rm{\lambda }}}}=\frac{{{{\rm{the}}}}\,{{{\rm{observed}}}}\,P\,{{{\rm{values}}}}}{{{{\rm{the}}}}\,{{{\rm{expected}}}}\,P\,{{{\rm{values}}}}}$)^72^.

### Candidate genes and functional analysis

The region within a 1 Mb region centering each significant SNP was defined as the QTL region. The candidate functional genes were searched within the identified QTL regions on the pig genome assembly 11.1 (https://asia.ensembl.org/Sus_scrofa/Info/Index). Then, the biological functions of these candidate genes were investigated on PubMed (https://www.ncbi.nlm.nih.gov/pubmed) and the reported literature. For functional annotation, GO analysis was performed on DAVID Bioinformatics Resources (https://david.ncifcrf.gov). The Fisher’s test was used to assess the significance of the determined enriched terms^73,74^. Enriched GO terms (P < 0.05) were selected to investigate the genes involved in biological processes^75^.

### Reporting Summary

Further information on research design is available in the Nature Research Reporting Summary linked to this article.

## Supplementary information

Supplementary Data 1-2

COMMSBIO-21-0181B-nr-editorial-policy-checklist

Supplementary Information

Description of Additional Supplementary Files

Reporting Summary

## Data Availability

The datasets supporting the results of this article are included within the article. All genotypic, phenotype data, and supplemental material were deposited at the figshare repository (10.6084/m9.figshare.12611786). All other data are available from the corresponding authors upon reasonable request.
