# Supplementary material for: A combined GWAS approach reveals key loci for socially-affected traits in Yorkshire pigs
Source: Commun Biol. 2021 Jul 20;4:891. doi: 10.1038/s42003-021-02416-3 (PMC8292486; doi:10.1038/s42003-021-02416-3)
Supplement: Supplementary file 3 — Supplementary Information [file 42003_2021_2416_MOESM3_ESM.pdf]

Supplementary information

A combined GWAS approach reveals key loci for socially-affected traits in Yorkshire pigs

Pingxian Wu [1,†], Kai Wang [1,†], Jie Zhou [1], Dejuan Chen [1], Anan Jiang [1], Yanzhi Jiang [2], Li Zhu [1] , Xiaotian Qiu [3], Xuewei Li [1], Guoqing Tang [1,*]

[1] Farm Animal Genetic Resources Exploration and Innovation Key Laboratory of Sichuan Province, Sichuan Agricultural University, Chengdu 611130, Sichuan, China

[2] College of Life Science, Sichuan Agricultural University, Yaan 625014, Sichuan, China

[3] National Animal Husbandry Service, Beijing, 100125, Beijing, China

* Corresponding author: Guoqing Tang, tyq003@163.com.

† Co-first author: These authors contributed equally to this work.

Supplementary Figures 1-7

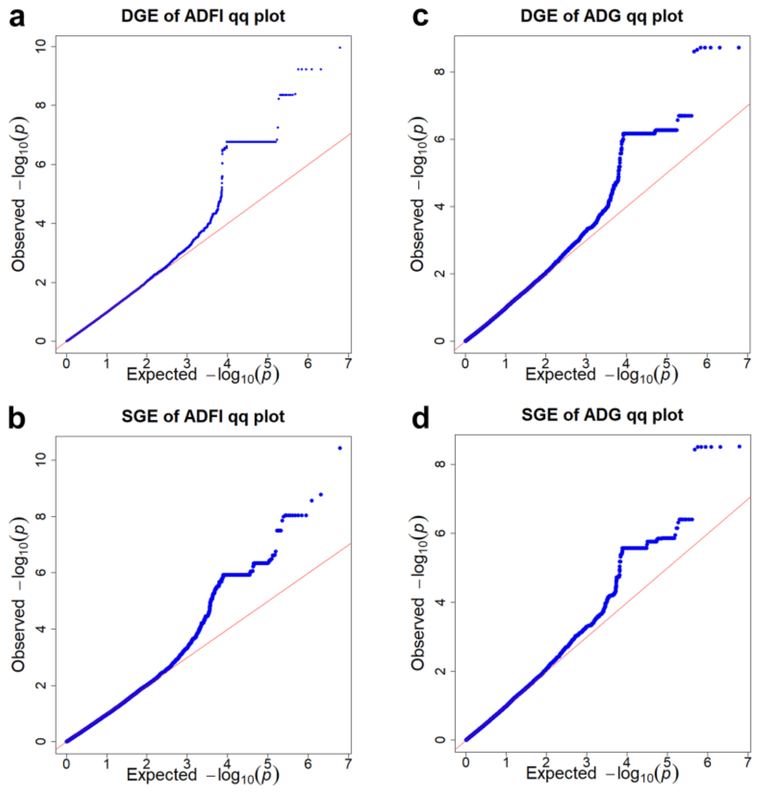

Supplementary Figure 1. Quantile-quantile (Q-Q) plots of single-locus genome-wide association studies for DGE and SGE. The Q-Q plots show the observed $-\log_{10}$-transformed *P-values* (y-axis) and the expected $-\log_{10}$-transformed *P-values* (x-axis). (**a**) for DGE of ADFI; (**b**) for SGE of ADFI; (**c**) for DGE of ADG; (**d**) for SGE of ADG

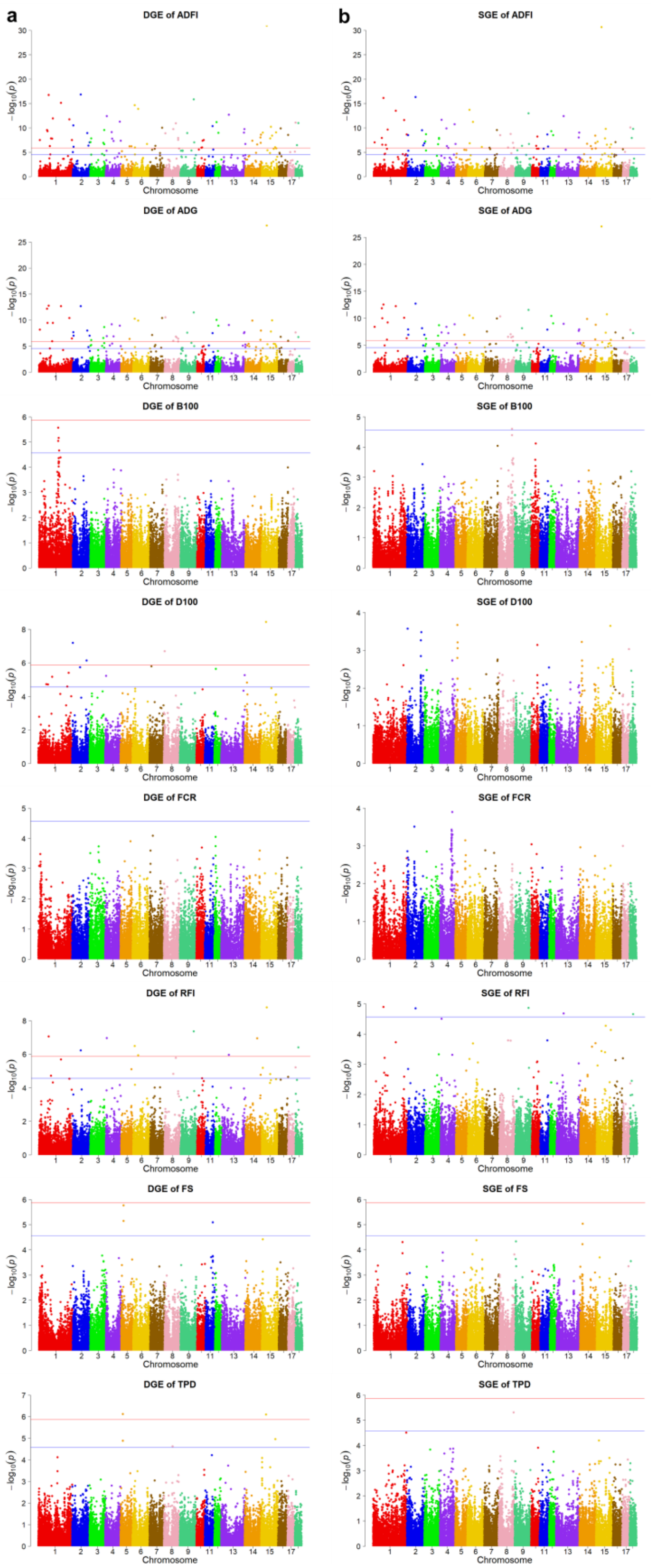

Supplementary Figure 2. The chip-based GWAS results for DGE **(a)** and SGE **(b)**. In the Manhattan plots, the −log10 (*P-values*) for SNPs (y-axis) were plotted against their genomic positions on each chromosome (x-axis). The horizontal red and blue lines indicate genome wide significant level (0.05/36,969=1.35E−06) and suggestive level (1/36,969=2.70E−05).

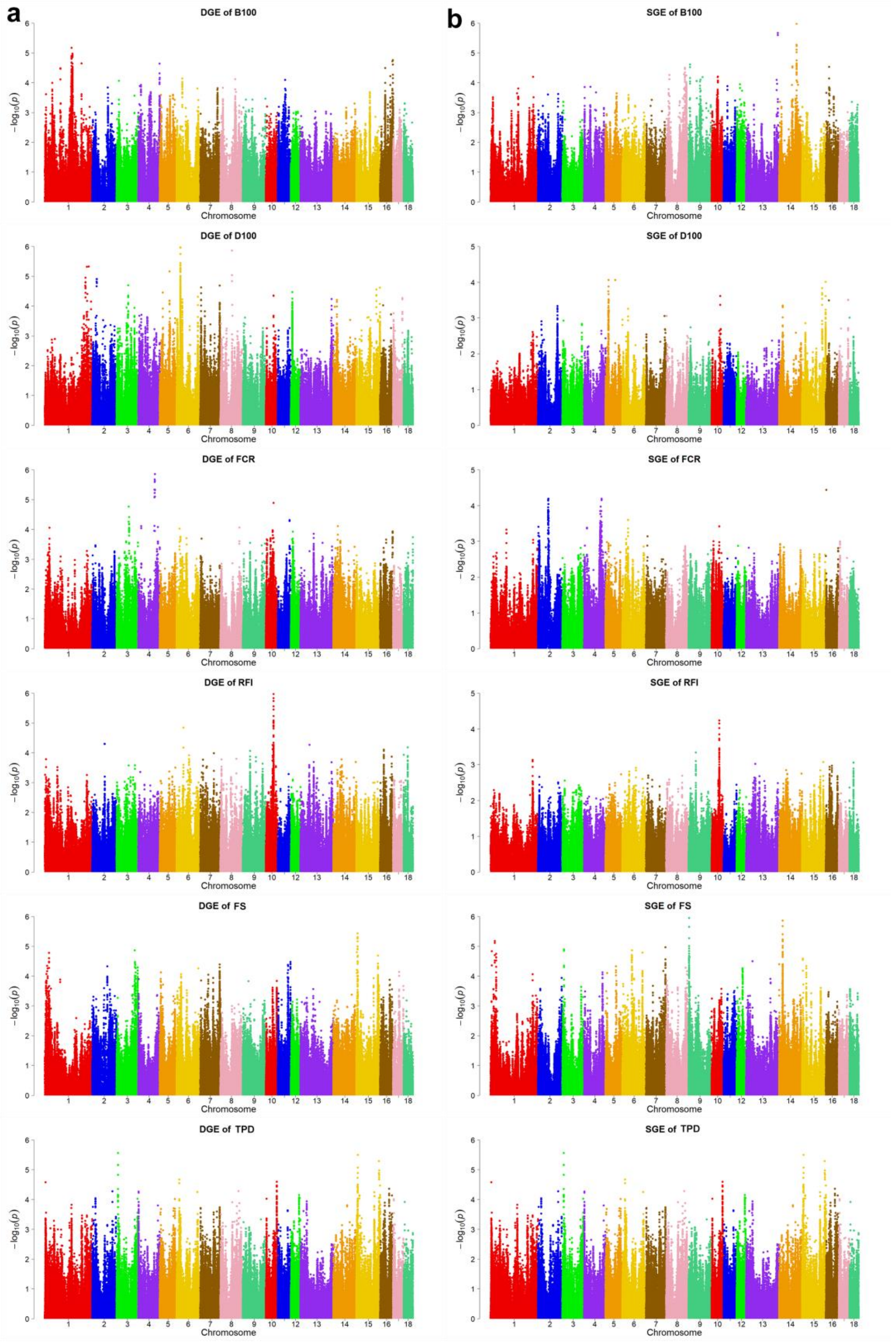

Supplementary Figure 3. Imputed-GWAS results for DGE (**a**) and SGE (**b**). In the Manhattan plots, the −log10 (*P*-values) for SNPs (y-axis) were plotted against their genomic positions on each chromosome (x-axis).

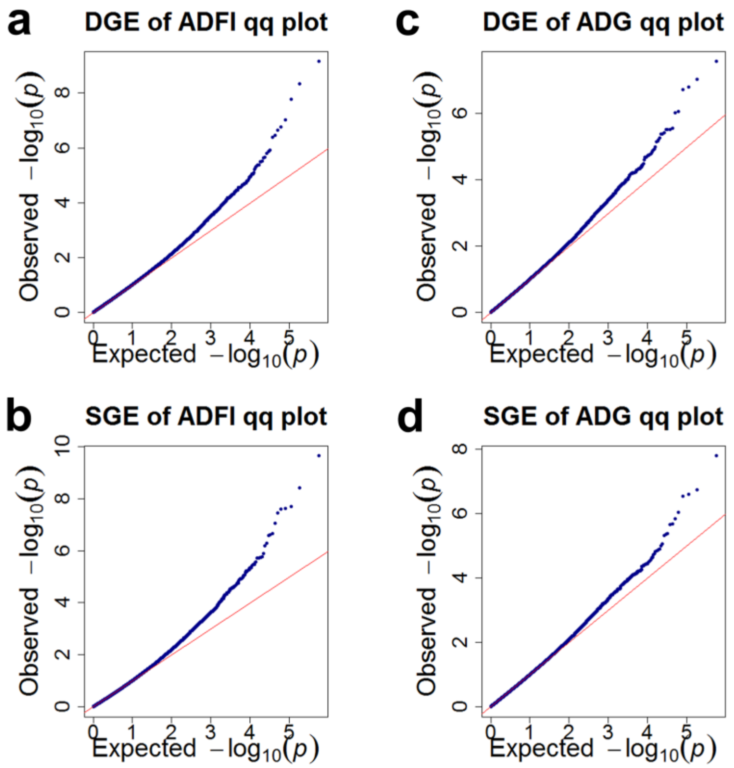

Supplementary Figure 4. Quantile-quantile (Q-Q) plots of Haplotype-based genome-wide association studies for DGE and SGE. The Q-Q plots show the observed $-\log_{10}$-transformed *P-values* (y-axis) and the expected $-\log_{10}$-transformed *P-values* (x-axis). (**a**) for DGE of ADFI; (**b**) for SGE of ADFI; (**c**) for DGE of ADG; (**d**) for SGE of ADG

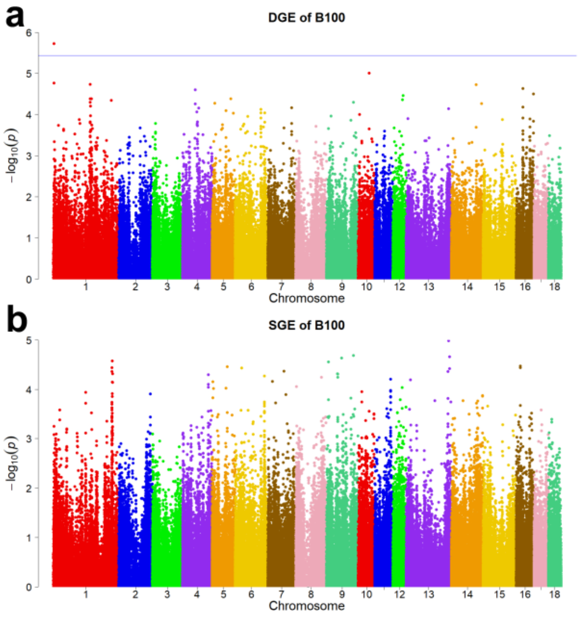

Supplementary Figure 5. Haplotype-based GWAS results for DGE and SGE in B100 trait. In the Manhattan plots, the $-\log_{10}$ (*P-values*) for SNPs (y-axis) were plotted against their genomic positions on each chromosome (x-axis). The horizontal blue lines indicate suggestive (3.64E−06)

level. **(a)** for DGE of B100; **(b)** for SGE of B100

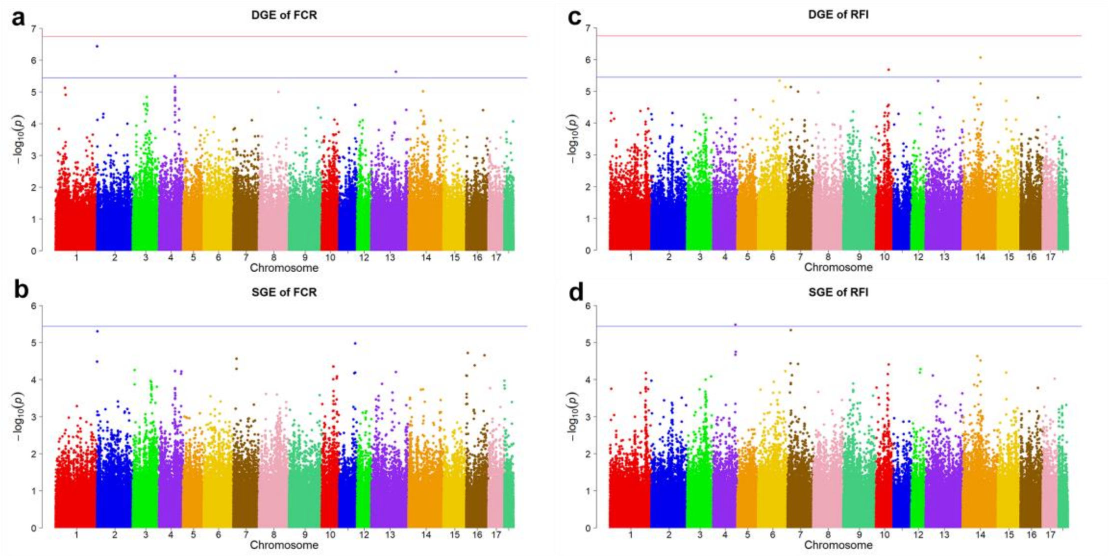

Supplementary Figure 6. Haplotype-based GWAS results for DGE and SGE in FCR and RFI trait. In the Manhattan plots, the $-\log_{10}$ (*P-values*) for SNPs (y-axis) were plotted against their genomic positions on each chromosome (x-axis). The horizontal red and blue lines indicate the genome-wide (1.82E−07) and suggestive (3.64E−06) level, respectively. **(a)** for DGE of FCR; **(b)** for SGE of FCR; **(c)** for DGE of RFI; **(d)** for SGE of RFI

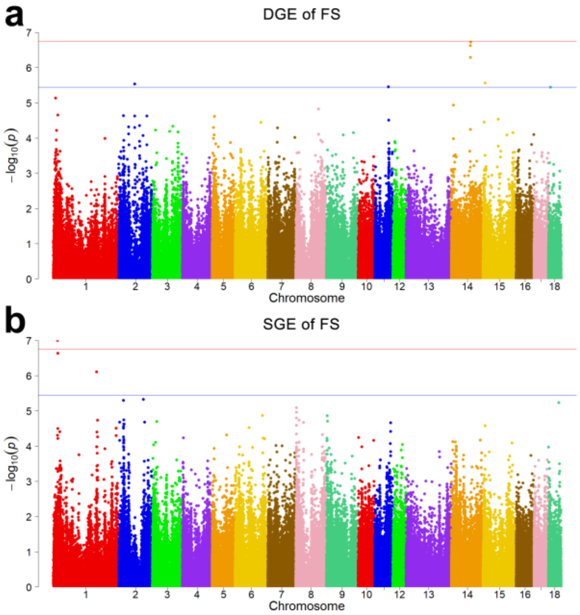

Supplementary Figure 7. Haplotype-based GWAS results for DGE and SGE in FS trait. In the Manhattan plots, the $-\log_{10}$ (*P-values*) for SNPs (y-axis) were plotted against their genomic positions on each chromosome (x-axis). The horizontal red and blue lines indicate the genome-wide (1.82E−07) and suggestive (3.64E−06) level, respectively. **(a)** for DGE of FS; **(b)** for SGE of FS

Supplementary Table 1. The overlapped SNPs in chip-based GWAS and imputed GWAS (P < 2.70E-05)

Trait	Chromosome	Position (bp)	P-value in chip-based GWAS	P-value in imputed GWAS
DGE_ADFI	1	282662716	1.72E-12	9.96E-06
DGE_ADFI	6	46465683	1.22E-14	3.18E-07
DGE_ADFI	8	79506715	1.86E-10	1.66E-05
SGE_ADFI	1	282662716	2.46E-12	3.80E-06
SGE_ADFI	6	46465683	5.64E-12	4.46E-07
DGE_B100	1	183316056	6.82E-06	1.38E-05
DGE_D100	1	270327686	2.60E-05	1.58E-05
SGE_FS	14	23840807	9.19E-06	6.25E-06

Supplementary Table 2. Haplotype-based GWAS results (*P* < 1.82E-07) for DGE in Yorkshire pigs

Trait	HapL #	SSC	Position	Haplotype*	Haplotype frequency	Haplotype effect	*P* _value	Candidate gene
DGE_ADFI	HapL1834	1	275922211-276425999	CCAAGAGGAATCCCTTTTTTTTACGCGGACCCGAGGGGCCGTGCTCTACGGCGTAGTACTGGTAATTGGTGTTGCAAAACATCCGGCTAGGAAATGGTCGTTGAAGTATCTTAACGGGTATTCGGACTAGTGTACTGATG	0.01	-0.24	1.69E-08	
	HapL1412	6	18635874-18635895	GGG	0.05	-0.12	9.22E-08	MT3
	HapL1193	10	61915547-61920091	GAAGCTTATACGGCAAATGTGGTGCGCTGG	0.02	0.19	4.46E-09	
	HapL910	12	7390616-7394637	TTTGTGCAACCATTACTCGAGCAAGATGGATTTAACGGCCTTACCGTTCTAACG	0.03	-0.2	6.92E-10	
DGE_ADG	HapL910	12	7390616-7394637	TTTGTGCAACCATTACTCGAGCAAGATGGATTTAACGGCCTTACCGTTCTAACG	0.03	-0.15	2.64E-08	
	HapL1834	1	275922211-276425999	CCAAGAGGAATCCCTTTTTTTTACGCGGACCCGAGGGGCCGTGCTCTACGGCGTAGTACTGGTAATTGGTGTTGCAAAACATCCGGCTAGGAAATGGTCGTTGAAGTATCTTAACGGGTATTCGGACTAGTGTACTGATG	0.01	-0.19	9.02E-08	
	HapL1193	10	61915547-61920091	GAAGCTTATACGGCAAATGTGGTGCGCTGG	0.02	0.14	1.59E-07	
DGE_D100	HapL1601	2	146426523-146748946	TATGGGGAGCGGGGCTTCATACTAAATTTTGAGCAGCCTATTGCAGCAATTCCAA	0.02	18.37	2.56E-08	
	HapL36	3	2979742-3015195	AGCAAACA...GGGGGCAA	0.02	16.09	4.33E-09	SDK1
	HapL213	3	3169344-3177734	CGTGGTAAAATCTCGATTGACATGTCAGTTGGTTATTCGAACGATGTGTTCTTATAAGTTAGTGTTCTGCTTAGTCTGTTT	0.02	14.69	4.12E-08	SDK1
	HapL181	7	51827448-52540567	ACACCGACTACAAAAC...GCAGGTGTAGA	0.01	22.56	9.58E-10	PDE8A, TM6SF1, BTBD1, C15orf40, HOMER2, WHAMM, AP3B2, CPEB1
	HapL759	7	106237871-106507416	TTAGGCTA...GGTTGAAC	0.04	11.84	1.06E-07	
	HapL762	7	106605539-106882656	AACTTGCA...ATAAGCCT	0.02	14.48	9.27E-08	
	HapL2197	8	38588797-39104506	CACGCTGA...AGCCCATA	0.02	17.47	3.95E-10	FRYL, OCIAD2, CWH43, DCUN1D4
	HapL2206	8	40617403-41064266	GCGCCATC...AAACTTGG	0.02	20.54	3.54E-09	CHIC2, GSX2, PDGFRA
	HapL2208	8	41096224-41237766	AAGTGGGC...TAATAATA	0.03	12.67	4.15E-08	
	HapL78	12	13926447-14221622	TTAAATTC...GTGTGAAC	0.01	16.68	3.07E-08	NOL11, BPTF
	HapL1399	13	202389100-202461016	CATCAGACGTCTCGAGGCATTGATGAGCTCCATAAGTCCAGGCGCA	0.02	16.96	5.79E-08	
	HapL1066	16	16761589-16929373	CTTGAAGT...CGTGCTAC	0.01	21.11	1.00E-07	
	HapL1069	16	16941814-17012360	GGTACAAA...TCAGAGTT	0.01	21.11	1.00E-07	
	HapL1073	16	17014404-17022242	AGACTGCTAAACGGGAGTACCCTCACGTATATCCCTTATCTTCAAAGAGAAGAGCTCTTAACTTACACAAACCGATGTT	0.01	21.11	1.00E-07	
	HapL1073	16	17014404-17022242	AGCCTGCTAAAAAGAATTTCTCCTATGAGTGCGTTGCCGTGCTCTGGGGAAAAGCACCTAGTCAATGTGGGTTTGGACC	0.01	21.11	1.00E-07	
	HapL1078	16	17024403-17033863	AAGGCTTGTTTAACGGGATAAGCCAAGTCAGCGTTTGTCCATCGGCCTAATATATA	0.01	21.11	1.00E-07	
	HapL1078	16	17024403-17033863	AAGGCTTGTTCGGGAATACGGAGTGCCGAATTCCCCACCGGAGAGTTCGGAATAAG	0.01	21.11	1.00E-07	
	HapL1083	16	17035724-17047602	AGAGACTGAGCGTAGTCTTGGCGGCGTGGGAA	0.01	21.11	1.00E-07	
	HapL1085	16	17048802-17059077	TGAAATGTCCACCTACGTTATATCATCTATCATCCA	0.01	21.11	1.00E-07	
	HapL1086	16	17059099-17075409	TAACGTGCTCTCTGATATGAGATTATCAGCTCAGTTTCAAGTATTCCCAGATGTCGCTAT	0.01	21.11	1.00E-07	
	HapL1086	16	17059099-17075409	CAGTCTTTTCCCTGAAAAGACACTATCAGCTCAGTTTCAAGTACTCCTAGATGTCTTTAG	0.01	21.11	1.00E-07	
	HapL1087	16	17075616-17093253	ACTCATGC...ATAGGTTG	0.01	21.11	1.00E-07	

	HapL1095	16	17106392-17116817	ATTGTAATTCAGACCATGTCTGGTATGCTTGGAGTGAAAGGTTGTTACTCTGTATG AACGGATTGTAGTT	0.01	21.11	1.00E-07	
DGE_TPD	HapL689	4	130340342-130390531	TATGATTT…TAAGGTAC	0.02	6.64	9.39E-08	ZNHIT6

DGE_ADFI, direct genetic effects of average daily feed intake; DGE_ADG, direct genetic effects of average daily gain; DGE_D100, direct genetic effects of days to 100 kg; DGE_TPD, direct genetic effects of time in feeder per day; HapL #, Haplotype locus; SSC, Chromosome; *, The detail haplotypes were listed in S2 Table.

Supplementary Table 3. Haplotype-based GWAS results (*P* < 1.82E-07) for SGE in Yorkshire pigs

Trait	HapL #	SSC	Position	Haplotype*	Haplotype frequency	Haplotype effect	*P* _value	Candidate gene
SGE_ADFI	HapL1832	1	274735024-275588282	TCCTTTCAAATAAGTACAGCGAGAACTCATGGG	0.02	0.05	3.55E-08	
	HapL1834	1	275922211-276425999	CCAAGAGG…TACTGATG	0.01	0.06	2.23E-10	
	HapL1412	6	18635874-18635895	GGG	0.05	0.03	1.99E-08	MT3
	HapL1628	6	25497660-25535657	AAGTATGCACACATCTGCGCTGTGTTTCCTCGCATCGCTCAAATTCACGATCAGGCCCG TCTGCAACATACAGTGGCAACTTTTAATAGGGAGGATACAGCATCGGAGCGTCGG	0.02	0.04	8.71E-08	
	HapL1051	7	123767361-123832312	CTACATAGCCTTCATGCTGTGCTAG	0.03	0.04	2.49E-08	
	HapL1193	10	61915547-61920091	GAAGCTTATACGGCAAATGTGGTGCGCTGG	0.02	-0.04	2.33E-08	
	HapL910	12	7390616-7394637	TTTGTGCAACCATTACTCGAGCAAGATGGATTTAACGGCCTTACCGTTCTAACG	0.03	0.05	3.88E-09	
SGE_ADG	HapL910	12	7390616-7394637	TTTGTGCAACCATTACTCGAGCAAGATGGATTTAACGGCCTTACCGTTCTAACG	0.03	-0.06	1.60E-08	
SGE_D100	HapL1601	2	146426523-146748946	TATGGGGAGCGGGGCTTCATACTAAATTTTGAGCAGCCTATTGCAGCAATTCCAA	0.02	7.34	4.87E-08	
	HapL36	3	2979742-3015195	AGCAAACA…GGGGGCAA	0.02	6.3	1.27E-08	SDK1
	HapL213	3	3169344-3177734	CGTGGTAAAATCTCGATTGACATGTCAGTTGGTTATTCGAACGATGTGTTCTTATAAGT TAGTGTTCTGCTTAGTCTGTTT	0.02	5.91	5.79E-08	
SGE_FS	HapL2681	1	20356655-20389162	GACTGTTTGAAGTTCACTC	0.02	1.31	1.00E-07	

SGE_ADFI, social genetic effects of average daily feed intake; DGE_ADG, social genetic effects of average daily gain; DGE_D100, social genetic effects of days to 100 kg; DGE_FS, social genetic effects of feeding speed; HapL #, Haplotype locus; SSC, Chromosome; *, The detail haplotypes were listed in S2 Table

Supplementary Table 4. Haplotype-based GWAS results for DGE and SGE (1.82E-07 < P < 3.64E-06)

Trait	Haplotype #	Chromosome	Position(bp)	Haplotype	Bonferroni corrected P-value
DGE_ADFI	1698	9	20052831-20057732	TATAGTTTGCGTACATGTTCGCCCTTCCGCCTGGAGTTGC	1.75E-07
DGE_ADFI	1051	7	123767361-123832312	CTACATAGCCTTCATGCTGTGCTAG	2.26E-07
DGE_ADFI	1832	1	274735024-275588282	TCCTTTCAAATAAGTACAGCGATGACAGCTCCTGCGCTTAGGCGGCAAAACGCGTGCGCTACATTGCACTGCAAACGGCTGGGTGGCACAAAGGAATATTGCTCATACTGGGGGCCACTCGACTCACGGTCGTACGAATAATCAATGCCCCATAGTGTGCCAGGTAACTTATGTCGCTCCGGCCAGTGCTTGCTGGAGTGCCGTGTCTGCCGTGAGCTGCAAATACGATCAAGCAGGTTACTTCGGGTGGATTCGGACAAGTGATGATATTCATACTTCGTGCCAACATTCACCGTGCCCCCGACCACCGCCCAGAATTACGAGAAAGACGAGAGGGTAATTTAAGAAACATCTCGGACAATTCAGTTCCTATTCCTTGGGCACCACCGAGTAGACGGCACTGTTTTTCGTCCTAGGTCTAGCAAGGAGTCCAAGGCATCAACTGACAAACTGATTCATCTGTGAGTGAAATATGATCCTGACGTTCTTGAGATGAACTCATGGG	3.43E-07
DGE_ADFI	379	12	3928649-3960434	CTCGATCTGGAGGTGGGACACGCGAAAGGGTTCGCGCGCCACAGGATGCGGTGTTATCGCAGCTGGCGCTAACGGAGCCAGGCCGCCGGGAATAATTAGTCCCGACACAATGTCCAAATCGGCCGGACAGTTGGCGCCAGCGGGACCCACCAGTAGCACGCCCTGGTGCGGGGAGCTGCCCCCCGCCAGCCTGACCAATTCATCGATTTAGCTCAGATTCAT	4.08E-07
DGE_ADFI	2009	6	46856839-47856839	ATTAGCTTCTTCGGCCCGACCTCACCTAATTCTTAACAATCAAGACTCTTCTATTCAGAACGAGAGAAAGCGGATCCAGTGCACTTGGCAGTAGTTCCGTTTTCTCGATAGGGCCTTCTTCTGCAAAACTCTGTAGGTAAATAGCCTCTGCGAACGGTGGTGCCACACCACGTTTTTGCATAGAGGGTCTGAGTCGCCGTCAATCGCGCCGTCTTAGCCATGTTAATCGTCATGACGTCGCGCGACTCAATTGCCACGTAACCTCCTACTTCGGGCGGCAGCGATTGCACATTGAAATGTTTGCGCTTCTGTATGCTCATAATATTCGGTTCCCATTATTGTTTGATCCATTAATTGTAGAAGATGCTGATAAAGCGGATTGGTCATTGAGCAGCGAGAATTGGGGCTATGGGAAACAACTGAGAAAAGCACCGTTAATCTCGTGTAGCTTGACGGAGTTGGATTTATCATTGTGGCATACTACCATTAAAATCGTTCTCCGGACTTGTTCTTGGCGTACTGAGCGCCGTAGGCGTCCAGTAGTAGGGAGTACGTACAAACGAATCCTAGATCCCGAGGAGGTCCTAAGCCTCAACTTCTGAGAAGGGGCATATTTACCTCTCGGCGCAAAAAAATTGCCGGTGGCAGACGCCATGCCCTGCATACGAGATCACGCCATCCACCTAACTCAGACCTCGGCCGAGGTAACTGGCGGGGGGCAAACCGGTGATAGGTCTCATTCTAGAGCACAACGTAATGATCACGTCTGGCGTAAGGGACATACACCGCGAGGCCGCCACACCGCGTCAACTCACTAAGAATAGGCTTATACCTAATTGCCTGACATTTTATGTTAGGAAGGGTACCTTTGGCTACAATCGATCAACGAGTGTGAGCAAGGCCTTTAAAGTCCGATCTGGACCCATTCACAAATCTGATCTGCGATTGCTCGAGTGACGGAGT	1.19E-06
DGE_ADFI	14	12	7980942-8179537	ATTTAAAACTCAGGTCACTGTCACCTAATCCCCACACAGCTCACTGCCCCGCTTGCTCCCCCCACCTACGTCCAAACCTGGGCTTGCGTTGTTATTGTTGTATTACCGGAAACAGCAGGGATTGGCAGCGCCGATTTGAAACACCGTGGGTACGTTGCTCGGGAGATGCACATTTCCCCTGCGCCGCTAGTTGCGGCACCTACACGTAGGCGCAGGGGCAAGTCGCCACTACCGTGCCAACGAGGCGACCATCTCGGGACCCGATAATTGTGCTTGTGTTCACTGCACGGGCCACCCGCCCCAAAATACAAGGGGGGCTTCGTGAACCCCCAGTGCAACCCTTCCACTCCCACAACCGCAGCGCGGCCGGGGGGCTTCTGCCAGCCGTCTGCAGCATCCAGGGATGTATGTCATTAGCTGAAGCGGGCCGACAGGATAGGTGTGCCTCACACTTCTGGGGGGCCCCAGGTGAGGGAATGCCGGCGGGCTGGACCA	1.37E-06
DGE_ADFI	1044	7	123745918-123759074	TAGCGCAATGGGCGGTCTTGTGCGGGTGGTAACGGGGATTCGACACACCCTAGGCTGTTGGCCAT	1.60E-06

DGE_ADFI	1966	14	28803917-28855000	GACGGTACGACTGCATGTAGTGATGTCATCACGTGTAGTCGCCGTTCGCAAGGCGAATTGGCTGCGTCCACTGCCTCTGTGGCATAGACGGATTGAGGCTAGGTGGTAGCTGGCAGGAAGCTTGGCCCCTAGTGATTCAATTTGAATTATTCCGTGATTGAACCA	2.22E-06
DGE_ADFI	1604	2	146870763-146878610	GGTTGGACATCAACTCGGAAGATTG	2.36E-06
DGE_ADFI	272	10	6359863-6360406	CCCTA	3.10E-06
DGE_ADFI	32	8	79508901-79509079	AAATAAC	3.23E-06
DGE_ADFI	32	8	79508901-79509079	CGGGGCT	3.23E-06
DGE_ADG	1832	1	274735024-275588282	TCCTTTCAAATAAGTACAGCGATGACAGCTCCTGCGCTTAGGCGGCAAAACGCGTGCGCTACATTGCACTGCAAACGGCTGGGTGGCACAAAGGAATATTGCTCATACTGGGGGCCACTCGACTCACGGTCGTACGAATAATCAATGCCCCATAGTGTGCCAGGTAACTTATGTCGCTCCGGCCAGTGCTTGCTGGAGTGCCGTGTCTGCCGTGAGCTGCAAATACGATCAAGCAGGTTACTTCGGGTGGATTCGGACAAGTGATGATATTCATACTTCGTGCCAACATTCACCGTGCCCCCGACCACCGCCCAGAATTACGAGAAAGACGAGAGGGTAATTTAAGAAACATCTCGGACAATTCAGTTCCTATTCCTTGGGCACCACCGAGTAGACGGCACTGTTTTTCGTCCTAGGTCTAGCAAGGAGTCCAAGGCATCAACTGACAAACTGATTCATCTGTGAGTGAAATATGATCCTGACGTTCTTGAGATGAACTCATGGG	1.92E-07
DGE_ADG	1467	4	17119823-17160785	GATTTGGCGGACAGCGTATCTCAGAAAGCCAGAAAATAGACACAAGTTACTGTCGTCGGCCAAAACAAGTGGGGACGGGTACAACAGGATGAGGGCAATCACCGTAACGACAGATAGTCTGCGATTCCGGAAAAGCCGTGCTGTTTCCTACTTCCAAAGATAGAACGTGCATAAATTTGGTCTCCT	8.82E-07
DGE_ADG	1698	9	20052831-20057732	TATAGTTTGCGTACATGTTCGCCCTTCCGCCTGGAGTTGC	9.42E-07
DGE_ADG	1051	7	123767361-123832312	CTACATAGCCTTCATGCTGTGCTAG	2.73E-06
DGE_ADG	1745	1	263897101-264896721	CTAATGTTGAAACACACTAGATGGAGCACTTAATACCGAAGCCAAGGGCGCCGGCACATCGAGCCGAGGGCTGCTGCGAATGTGGGACCCCTCTGACTATAGGACATCCTGCCGGGGTGCCATGGCATACACCGTCGTCCAATCGCCGACCCCTACAAAGTTAAAGAGAAAGAGGATAGGGAATCTTGCAGACAAGACCGGTTGATCAGAAAACCTCTAGGGTGATATGACGGCGATTCCACGGCGGGCTGAGACGGATGCCCGACTGTCAATCTAGTTTAACTTCGCACATAGCTACTGTGAGTGACTCACCTTGAAAACTTACTATTATAAAAAGTGATAGACGAAGCGTTTAGCAGCGCGTAGAACGGAGTTCATTGCGATAATCATTAGTAGTCAGGCAAGTACGTTTTAGCGTGCACGTTTATCAAACAATGACCCAAATAAGGATTGAGATCTAAGTTACATAGGGTTTAAACGGGAGGCTAAATGCCGAAGGGCTTTGGACCGGGTCAGTCGTGTTCTAGCGGTTCACCTTATCCGTATTATTTTGACACGGCATTCGCTCGCGTGTCGAAGGAGATCACCGTTCACTAAAAAGCTACGTTTAATAAGAAAGGTAAACATTTGAATTACCTCACCGACATCGAAAGAGATCCCGTGACAATGTCTGTTGTGCTGCCTTAACGGCTCTTGGCGCGTCGTCACATTTGGTAAAGTTCGGAGAGGCCGTAGGTAATATCCGTAGCTCCTACAGGTGCTTCTCTACTGAGCTTTTGCACCCTAATACTGGTCATCTTCTATCTTTGGCCGTAGTCTGATTCTACTTATACTCTCACGCATGGTTTACATCCACCAACGTCGACAACTGTAGGATAGCTCCCCCGCTATGGTTAACACCAATCCGGCGGACACCCCAAACGGTCTCTCTTTACCTAGCTTAAGGATCGACATTCCGTTGTCAGGGTGTATCCTTCCCGATTAGGTCGGCTGTGCAGTCTATAGGACATGTGTTTAGCGGGGCAAGATAGTGCCATACGGACTAAGTTGTTCCCCTAATGATAGCCCGCTGTCTAAATTAGGTTGGCGTAGAACGTTTAGCGTGCAATCCAATTAGCTGACTGACACCCTGCAATGAGATAAGGAGCCTCCCCACCCTGCTTCACGTAGCA	2.96E-06
DGE_ADG	558	7	5811660-5837760	GGTACATCGCATGCACATGCTCCACGGATGCCCCAAGTCGT	2.96E-06
DGE_ADG	1412	6	18635874-18635895	GGG	3.06E-06
DGE_B100	2700	1	3465273-3672411	GGAAGCGCGGTACTGCTTTGGTGAGTTAACA	1.90E-06

DGE_D100	1405	13	202484133-202490268	CCCTTTCGACAGCAGGGTGCGATTGACCATCTATTCCGGAGGTCAGCTATGGCGGCTGTATACTCACAGCAGGTCAAGCGATCGCCTCTGACCGAACCAGGTATAATGCGAAAACGA	2.65E-07
DGE_D100	74	12	13675903-13848011	ACAGTTCTTAGAGACTGCGAGATTAGATGCCCTCAATGGATCTCGTAAATAAGACTTCCGAATAGCTTCAGTCTCCTGCGGAGTTTGCAAAGAGGTGGTGTGAATTATACCTCCCCTTAAATGACGACACTTTCACCCGGAGGATCTGACGCGAGGCTCTGGGCGTACCAACTCGCCAGTCTGGATTATGGTGCGTAATTGCCGCCTCAATGCATCCGATTTACCGTTCGGGGCATCAAAATCAGCTGGCCCCGGCACAGAAACTATAATAGCAGTCGTGCTGTCTTTAAGAACTCGTTGCTACGTGCCTGGTCCCGGCTCTCGATGATCGCGAAGCACCCATTCCTCTGTTGGAGAGGTCATACGAGCACATGACGATCAATTCTACTCTTCGTCCGTACATATTTGTATGAATGGCCTCTGGCACCTTAGGAAGTACGGATGTGTCAAGCAGAAGTGTCTGATTTCGAGGGGCGGGGACGAGCAATCTGATTGCTTGAAGAGCATGTAGGTCGGATTCATGTTGTCGCCAGCCTTTGCCACTGCTCGAAGTTCGGAGAGGTGTAACTTTGGCCTCACCACCAGATAACTTGGGTGTTGCATGTGTTATGGGCAATGGCGCCAACTTGTGGTTGCGCCCAACTTGACACCTCGGACTACACCCA	2.98E-07
DGE_D100	1956	1	282214916-282251616	CATAAAAAAGAAGGATAAGTTAAAAGAT	3.32E-07
DGE_D100	1368	3	13923967-13998209	TGTTTCGTCAAAGGGAAGGGAGAATGAGGCTGCA	5.05E-07
DGE_D100	37	2	39272553-39272985	ATAG	6.19E-07
DGE_D100	1736	14	2917686-2921237	GAAAAATATCACAGGGGATAA	6.68E-07
DGE_D100	1158	6	7169389-7175723	ACTTTTCCCCCTCCTAGTCGACTTCGTGACGCACGTGCAGGGGTTCCACCGGGGGAAGCGCCCC	9.62E-07
DGE_D100	2186	8	37716770-37756108	GATTAGACTCACTCCTTCACGAACCCGTTTGGCGTCGTAATTAAAGCCCAGACTTTACGATGTCGATGTCATTGGGCCGGCCTTATGGCTCTTTTCAGTAT	1.02E-06
DGE_D100	2187	8	37756140-37951782	GGTTTCATCGCACCCGACTTTATCGTACTGTCAAACACGAAGAGCTTCCCGTTGGATCTGTGGAACGACAGATGGGAGGCAAATTCACATATACGAGCTCGACAACTGATCGAATGTTTGTGCTGCGCACAGGATAGTCTGTTAGTCTATTGGATGGTTCCCACAAAACCAAGGATATGTCGAGTATCTATGGCCTCCCGTGATAATTTTTTTAGGTACTAGCCTTAAGACCACTTCTGTTGTTAATGGATGAAGTACGCAATTCCTCGATGCATGTTTAGAGGATGTGCCGCTATCATTCCGACCGACGAAAGTGAACCGAATAAGGAACAAACTTCACTAGAACGAGCTAGCGGGTAACTTGAGTAAATATAAGAGGCATAGTTTTTATCCCGCATCGATAAAAGAGCAGTCCGATTCAATGGGGTT	1.02E-06
DGE_D100	1834	1	275922211-276425999	CCAAGAGGAATCCCTTTTTTTTACGCGGACCCGAGGGGCCGTGCTCTACGGCGTAGTACTGGTAATTGGTGTTGCAAAACATCCGGCTAGGAAATGGTCGTTGAAGTATCTTAACGGGTATTCGGACTAGTGTACTGATG	1.12E-06
DGE_D100	1094	16	17105002-17105583	GAGA	1.15E-06
DGE_D100	2203	8	40150641-40497989	CAAGCGCCCTGAAGGCTCAGGGCGCAAAGTGACACGTATGCACACTTATTGGCCACAACTTGGTTGGTAT	1.20E-06
DGE_D100	1861	13	218109226-218139214	CATTAAGCGAATCGTGCAAATGGAGCACCCTCGATATCAGCGATGGCTAGGTAATGTAGAAACCACTATTTGAAAATCCGTAAGCTG	1.75E-06
DGE_D100	1358	9	13517556-13518554	CGCTACTCGCTT	1.87E-06
DGE_D100	105	3	76448316-76705229	AACTAAGTTCCCCCCTCCGTGACGAGACGTTCCTACGTTGAACGGCATGAGAATGTCAAGTAGACG	1.89E-06
DGE_D100	1858	1	279103504-279164745	TCTGATAGACGCCGACGGTCTCACAGCAGGTAATGGAGATGGTCGTCCTCGGTAATATGTGTTCGGGATAGGTAGCCGATCTCTCGACAGACGTTGATGCATTCGTATTCAAGCATTTACCTCTTTGTGCAATCAACACTAAT	2.13E-06
DGE_D100	348	2	84020866-84064177	AGTGTTGGACAACCACATTCCGGCACCCGACAACAAGTCTATTTCGCGCTCCGACTATCCGATTAGATTTTGTAAACAACCCCTACGCGAGAAACCAGGCCCTTTCGTAGCGGCATCTCACAGACGTTATGAGCTGAACATTACTTGCT	2.14E-06

DGE_D100	1735	3	36042997-36645481	ACTGTCCACCCGTCCGTAGCGTTAAACTGGTAAAAGGCCCTTGGCAGCGCGAGGATTCTACCTGTCCGA AACGCCCCTTCGCGCCGCTTCGATTCTCTCCACAGTTGTTGTACGTTACATCCCCCACAAACCCTTCGTG GCGTGGCCTTTGGTTCAACGGGACCGTCTGGACAAGCTGACCCGCCTTGCGATTCCTGGTTGGAACGGT ATATGAGCCAGGCTCCTACGGATCTTCATGCCATTCAAGCACCCGCTAGAAGAGCTTTTGTATGCGCGG CGAGCGACTCTTGCAAAGGCATTCTCTTACCTTTGAAACCACCAAGTGCAAACATATACTAAACAGCAT GAAAAGCGTTTCATATGACCGGCGGAACCACTGGGGTGGATCGGTGACTTCCGATTAGCCTCATGAAG AACGTGCTGCGTAACTGCTGCTCGGGCTGCCCGAGCAGTCGTCCCACGGCCACAGCTCGGGGCGTGTG TGCCATATCACACAAAACATCTCTAGGTGTGTATCATACGTGTTGATCGGCGATTAATTTCAGATTATGC ACGGAGTTTGCGCCCTGGGAAAGATTATGGTCCTTGTTTTAAACGTTTGCACCCCAATTCTATGTTCCAC GACATTCCAATTCACGCTGGAAGCAGCCAGAGTCCAACTTTGCAATGCGTCGGCGCTCCACCCAAACGG AGCCTGGTTGTGCGGCGCGCTCCTTAATTCCAAACCGGGGGGCTTTATTCGGTTGTAACTGCTATGGTA	2.46E-06
DGE_D100	527	6	125046444-125064556	CCGCCGCATCCGAACAACGTAATACCATTGGTAGTC	2.51E-06
DGE_D100	1843	1	277789240-278310829	AAGCCGTGTTCACACCACCGCCTGTGTTGGGTCCGCCCGGTATTATCCAGTATC	3.24E-06
DGE_D100	2210	8	41241396-42238430	AATCGCAAAGCCGGCCAAGCGAAAACTGTCCGGAGGCAAGGGTGCATATCAAAACATCTACTGGTCTT GAGGGGTCACTAGCTGACCTTCGTTGAAGCAATTGTGCATTAGCAAAAATTTTAAGTCCGAGGTGAAGT AGGTTACTACAGTAGCGAGGTCGGGAACCTTCATTTACTGCTAGAACCACAACTCTCTATGCTGTCGTG GGCGGCATCATCAAACGGAATACACCGTCAACCCAACTGTGGGTCGATGGTCGCGTCTAGATGCTTGG TTTACAAGCGTTGCACATTGTAAGGACATTGTGCGGAGTTCACTGCTGGAGCCCCGTTAGCCCGAGTTG ATTCAAATTTATTTTCGGGTTCCTTCCGCCGGACAAAGCCCACTATGGCTCGACAGACACCACCGGTCGT ACGAGTCCAGCCAGCACTCAGTGTTTTATGTCCTGTTTTCTATGTCCTCCGACACCGCTCAACGCTAAAC TCGAGTCGGGGACCCCCTGTCCTGCTCCTGGCAATGTAAAGCTCCTGGGTGCTCCGACTCAGAAACTGC ACTTTGAGTGGCTGATAATAATATAGCAAACGATCCCGTATCGAACTCTACCGTGGGGCCCTTGCGAGG TAATAGAGTATGTGGGTGATACAGTGTGGCAGTTAGTCGTACCCTACTTAGGCGATGCCGATAGTGTCC TTATTACGACCTAATTTTAGCGGCTTACCTACTCCGTTTTCCAGCATCTTGAATAGTCGTGATCCACTAGA CTAAGGTAGAGGACACTTCGTGGGCTAGTCGTATGGACGAAACTAGCGTAGAAACGTCCGATCGATTA TAATGGTACAACTTCAATCTCAAACTATCTCAATACGGCGGTTACCTTCTATGGCATCTATGGACCTCGG CTACCAAACATCTGGACTATTGATTGTGTCCAGTTGACATGATGCGTAATGGCAACGCCATTTTCGTAA G	3.33E-06
DGE_FCR	2215	2	2439963-2489895	GAATAATGGTAAGCGATTGTTAAAA	3.73E-07
DGE_FCR	259	4	110009202-110011016	ACGACGCGTA	3.21E-06
DGE_FCR	861	13	172549773-172556636	GCCACGTATAGCCCCTACGATTACTTATTATTAATCAAAGTTGGACACACGTCTGGTAGCATCTAAGTCG AATGTC	2.35E-06
DGE_RFI	1032	10	51709270-51710687	GATTAGCGGCTGATCA	2.09E-06
DGE_RFI	262	14	66346656-66350307	GTAAGTTTATTATAGATTAGCCCCTCCCCCTAGAAGGAAGA	8.67E-07

DGE_FS	450	14	93788835-94078025	CAAAGCCCAAACTAGACAGAGCTTGGGAAGGATCTCGCGGCGGGGGTGGTCTAAATCCTCTATCGACGTTACGTGGACTGCGTACCGTCACAATCGGGTCGCGGGAGGTGCGTTTCGCACGTACAAAGCAACAATAATCGTTGCCCATTCAGGGGTGAAAGCTCTTTCAGTTTAGTTGGACAGCAAGCCCGTTTATTCATTTTTGGGATGAGTCGGGCCTTAGAAGCAAGCATTATATATTTCTTTTTTAATGGTGCCGTCCCAATGCTAGCTTCAACTTGAGAGTAATTCATGTTTTGTAGTCTTCTGCCAGGGTAATCATGACAAAATGAAATTGGAAATGGTCTCTGGACAGTCGGTTTGCGGTTTGTGCGTCACTGTCGCGGAGAGACCTCTAGTACAGGAAAGCTGGTATTCCAGCGCCCTGCCTAAGAGGTGGATAGGTTTGGGTACCCGGGCATTTAGATTAGGAAGACTGGCAAACGCCTCCCTAGAGCTGTCGCAAATGGTGATCTCTAACCATATAGTAGTTTAAATCACTCCCTAGTTGTCCAATATATGCTGTAAGGGTACGGGGGGTAGTGCTCTTCTGTGTAGCCTTTCGTGTACGCCCAGGTTGCCCTCTGGTCTCAATCTGAAATACAGCATAACTCGACGTGGCCGGCACGTTCTGCGTAGAGCACCAATGCTCAGCCAGAACGTGACTCGTAAGTACAGCGGGAGCGCATAAGCCATCCCGACGAGTTTAGGAAGATTGGCACGGCATTTCGACCTACGGCGGCACCATGATGGGCCCGGCTAGTTAGAATTCACCCCCCGCTCTGTAGCCGCCCTCCCTCTCGTAACGAGCCGTGATTAGGTACTCAGAGAGTGCAGCTCGTAACGCTAGACCGTCAGCTCCCCACAAGGCTTGGAATCACCGCCTGCTGGACTGTCCCTAGGCAGAAGAGGAAGTTCTTTCCTCGTCGTATTAACCACGGATGGAAGCATGTTCATGTCCGAGTAAATGGATGCTTCCTTATGACAGACTGTACCCTACGCTCGTTCTACTACGCTTCCGGACGACGAGGAAGGAGTACCCGCTGCGCCTCCCTCTGACAGTGGGCTTACTGCTACTCCAGCTTGTTGGAGGAGGAAATCGGGTTACGACTTCTTGGCGATCACTTGGCAT	1.88E-07
DGE_FS	440	14	92136331-92190618	TAAAGATCGAGAAGATATAAAAAAACATCCCTGTGAAAGGGAATACCGGTCCATTCGGCGACCTCGTGGGCCTTAACCTCCAGGGCGAATCAACCAACCTATCTCATACTGTGGTCACATTATGGCAGTTTGGTACGGGCAGAAGACCTTTTGCAGCTGTAAGTCTTTTGCCGAGCTCAATAGTTGAATCGTGCGAGTGCCGAGAGCGATAGGGGTACATATAGATCACGGGATTAACCAATACGCTCAGTGACTGTACAGATTGGAATAGTTACTAAGTTAAACCCAGTATCAGCAAGCCTCACCGGTT	2.39E-07
DGE_FS	425	14	92083574-92107595	AAACCCAGGTCAGACAGATAGTAAGGGCTAGTAATAGTTATGATGGTAATGAAGCGCAACCAGACAACCCGAGGGCTGTGGCTCACGCTCTGACTTTACTCTTGAGAGCAAAGTGGTGCTAAACATGGACTCAGTAGTGTATTGGCCATCACAGTCATTGCGAACAGTTCGAAGGCTGAAACCTTT	5.18E-07
DGE_FS	948	15	8389975-8390374	CGTG	2.79E-06
DGE_FS	323	2	76873073-77873073	ACACCAGACAATCTGCTCCCGGGAGGCCCCTCCCATTTACGTGACCAATTTTCTTTTACTCAGCACTAGTCCCGCAATGTGATGCGAAGATGTTCGATCACGTACCTAGACGCGAGTTAATCACGTTTCAAAAAGACCTTCGATCGGCAAGACGCACGTGGCAAAACGGCATGGATACCACAAAATCCATGATGCAAAGGGCAATCGCGTGCATAGCACGCCAGACTCGGACAACCGTATAAGCTGCTGGCCCCGTATACCACTTCCGACTTTCACCTTTTGTATGCCAATTGATATACAGCTTAGGTGGCGCTGCCGGAAGACGGCCTACCATAGGTGTCGAAGCAAGCCGCCGCCGACCTGTAGGTGCGATATCAGTAACCGCAAGCGATAGCCGCCGAAACATACGGTCCGTTATATAGGCTACGAGTTGGAAGACCACTCATCCATGATGAGAGTCACCAACTAAAAGCGTCCGGTGCCTCCCCCGATCGAGCTAATTCTCGCTGGCGGCACTCACAGGGAGCGGCCGAGGTAAATGTGCTACAAGGTGGTGATGAACCAGAAATGAGACATTAGGAGGCGCTGAGTAATAGTGGGTGGATGAGTTATTTTAGAACCAATAATATCGCCTTTAAAAATCATCCCAAGCTATTCGCGTGATCTATTTTGAAGTTGAAAAATTTGAGAGATTGTAGTTATAAATACATGCGTTGCAAGGTCTACTGGCTATAGACGTGGGGTCTCATCGCCTTTCCGCTGGCCCTTGCAAGGAGTCCGAAGAGGCTATAGCATATAATAGACTGGTGTCGATAGTCAGTGCCTTCCCCGGTCACAGACGGAACCTGGTGGTCAATCCGTGGCA	2.94E-06
DGE_FS	1377	11	66808079-66817247	CTCCGCATACTTTAGCAAGGCTGGACCACG	3.56E-06

DGE_FS	21	18	10403245-10508872	AATAAAATCTCGAATCGCGATTCCCATCGCATCATATTGTAGAAAGTCGGACACTTTAGGTTACCATAGACGCATTCTATTTCCCTATCAGCCACTTAGTCAAGACATGGGTGGACTAAGAGAGGCCTTAACCTAGATTAGGAGCACGAAAATATAGCATCAGTGTGCAGTCGGCCCGGCTGCAGAACCCAAAGCTGTTTGTCAACCATAAGGAGTATAATGTTGTTCTTCATTCCGCGAACGAACCACGAACGATTAGTCCGAAGATAAAGCATTCAAAGTATCTCCCACGTTTAGGGGTCCGCTTAGTGTGCATTGGGGGTTCGAAGTCCCGTTTGTTATGATGGACTTGATGAGTTTGTAAACAAGATCTTTCCCCTTGAAAAATTAGATTCGCCGACTGACGCAAGTTTGTTTTGGTGCCATACTATAGCTTTATATCGAATACTTCAGCGCCTATAGGTGCTGGTCCTG	3.63E-06
DGE_TPD	699	4	130607376-130620542	AACAACTCACGATCGAGCTAATCAAGGTCAACTTTGAAGTGTTGACATGGGTCTC	8.24E-07
DGE_TPD	339	7	75332634-75356235	GCCTCGTGACTTTTCTCTCAAGGTCGCAATATTGCAGCTCATCACCATCTATAACCATGA	2.41E-07
SGE_ADFI	1044	7	123745918-123759074	TAGCGCAATGGGCGGTCTTGTGCGGGTGGTAACGGGGATTCGACACACCCTAGGCTGTTGGCCAT	2.15E-07
SGE_ADFI	379	12	3928649-3960434	CTCGATCTGGAGGTGGGACACGCGAAAGGGTTCGCGCGCCACAGGATGCGGTGTTATCGCAGCTGGCGCTAACGGAGCCAGGCCGCCGGGAATAATTAGTCCCGACACAATGTCCAAATCGGCCGGACAGTTGGCGCCAGCGGGACCCACCAGTAGCACGCCCTGGTGCGGGGAGCTGCCCCCCGCCAGCCTGACCAATTCATCGATTTAGCTCAGATTCAT	2.50E-07
SGE_ADFI	1944	14	28407091-28411314	TCTCACCACTGATTATTTATCA	2.55E-07
SGE_ADFI	1698	9	20052831-20057732	TATAGTTTGCGTACATGTTCGCCCTTCCGCCTGGAGTTGC	5.09E-07
SGE_ADFI	78	12	13926447-14221622	TTAAATTCATTGAATCACCTCTGCCAATAATCGCAGTCAGAGCTACTCTGTCTTCATTGATTACCATCACTCTCGAGCCAAATGTACTACCACTCAGGGGGGCGATACCCTTAACACAGATGAATAGCATTGCCCAAAGAAGCCCGCTCTCTCAATAACAGACGACCTTGGCAAGTGAATTCAAGAGCCAACAGTTTCCAACTCGCTTGCTTCGCTTCTTTTATCCCAGTTCAAACTCGCGCGCGGCCTTTGCACCTCTTGAGCCATTTTGAAAAGTGAAACTATTGTGGTGCGCCTGTCTGTCCCGGAATTTTCTACTTTAGTCTCTTAGGATGGGACTAAGATTCCAATTACGCCGCTTGCGAAAGAGTGCCAACAAAACATCGATCAGTCAAGAGTGAGCGAAGCTATCGTTATCTTTACAGCTGCCGGAGGCGGCTGGTTTCGAGGGTTAACCGTTCCCCTGAACATGAGTAACTCTGTGCAGATCGCCAGAGGACGCTGGAGTGCCGCCCTGTGCGGGTCACGTACGTTGCTTAGCTGCTTACGTGTGATGGGGTTATTGACGCTCTTCCTAACCTGAAAGCGCTAATTAGTAGTTCACATCAGACCCATTTCGAAGCTGCCAACATATGATACCATTCGTCGGCACCCACGTCCGTGGTCTGGTGTGCCTAAAGAACAACGATTTGGCGGGCTGGGTGCCCAGGGGCGTCCAATGTATAAAAATCTAACGCGATGAAAAATAGTCGGGGTCTACTAAAGGCCGGAGCCGGGACACGGCCTGTTCCGTACTGTCTGAAGCAGTTTGGTGCGGGGAGGGTAGGTGGTTAAAAAGTAGATACAGTTCTTAAGGACTCATAAGCAGTGTGAAC	6.47E-07
SGE_ADFI	207	11	73129104-73139552	TCATACCCGTTTAGATCCGTCGCATAT	1.31E-06
SGE_ADFI	1110	9	141481657-141503604	AATCCATAGTACCTACTATGTCATGCCGTGTGCGTGTGTAGCAATATCCAAAATCGACCTCTCTTCTC	1.67E-06
SGE_ADFI	1837	1	276432474-276449735	TAAGATGTGGTGAGT	1.80E-06
SGE_ADFI	1792	4	42512125-42767763	TTAATGTTGTAAAATGGTTAAATCCGGCGTCGGAGGAGACCCACTATTTCCCTACCGAATTGAAAGACTGCTTCTCAGCAACCCGGTTCGCCACGCACAAGGACTTCGCGTTGGTTGTCTCCCAGCTGTCCGTGGGCTGGGGCATTGGGATCACTAGTTCGCCCATGCTAGCGTACA	1.85E-06
SGE_ADFI	224	3	1450656-1703835	TATAGTCCCAGACACATAATATTTATTCCCAGTGTTGGCATTAGTTTTATTCGTCATTTAATAGTGTACTACTTATTACGGTCCGTAGGGTCTAAGACTCTTATAATTATCTGTGATAACTTAATTTTTATCAACTTTTGATACTCTTATGGACTTATGTTTAAATAATAACATTTCGTCCCTTAGCACAGTCTAGTGCCCTCTGTCGATGAT	1.95E-06
SGE_ADFI	206	11	72908025-73128938	GTGCCAAGTACCGAACCTGGCAACTCACGTCTACTCAGGGGAGGGACAAGGGGTGAGCGACGCCTGCCATCTAGGGTTCGTCGGATACACAAAGATGGAAGATGGGCAAAGCTACCGGAAATGTAAAGCCTGCCACAGCGGGCGGGTCGAAGAGCACTCGACGCGAGCCGTTAGCCAGCGAACTTTGTGCGTACCCCGTAATTCTAAACTCCCGTTCCCTATTCCAGAGCAATGTAGTTACCCAACAGAGAGATTGTTCGTATCTCAAGACCATACAAGGCAGCCATATAGGTGTGACTAATGGCGAATATAAATATGCGGTGGTTCGGATCAAACCCCCCATATTCGTCACCAACTTATTATGAGAGTTTACGGAATTTATAGGCCTCACGTGAGGAACTGCTTTAGCAG	1.97E-06

SGE_ADFI	208	11	73139693-73171161	TCATGTCGACGAAATCTTGTGAGATAACAAGATGTGCATCAGTTATAGCCCATCCGTCACTATGCCATTTATACAGCTGCCAAGCTGGGCCGTGCCGTTGGACCACGTATTCGCATCCTCATCGGCCTTT	1.97E-06
SGE_ADFI	799	16	9132548-9184660	TATTGTCCTGTGTGTGGAAGCGCACTTAGCTACCATGCGTTACACTTTGTCGATTATCTGGTTTCGGATCAAATCAAGGCATAGTCCAAACGTGATTAAAGTGTTGTTTATCCCGCGGTT	2.74E-06
SGE_ADFI	760	9	121252007-121265189	CCTTCCTAACGAGATCCGCCTACATCGTAATAACTGTCTCCCAGCCACTTACAGGTACTATCTCTAGCCGACCAAAAGTCGAAAGATTAGAACTGA	2.79E-06
SGE_ADFI	1716	9	21178075-21291626	TTCTACACCCCTCGCAATAGTACTCTGTGACAGCCTTCGCAATTATGCTTTCAGTCTTCCCATCAGGATTATCTGCGCCCGGTCTTTCACTTGATGATTCGTCCTGATCGCCGACCTCTCGCGCGACCGGGCCCGGGTACGCGTGGGTAGTTTTCTTGTCATTACTACTCGCAGCGGCCCTCGCTTTCCGTACAT	3.31E-06
SGE_ADFI	2009	6	46856839-47856839	ATTAGCTTCTTCGGCCCGACCTCACCTAATTCTTAACAATCAAGACTCTTCTATTCAGAACGAGAGAAAGCGGATCCAGTGCACTTGGCAGTAGTTCCGTTTTCTCGATAGGGCCTTCTTCTGCAAAACTCTGTAGGTAAATAGCCTCTGCGAACGGTGGTGCCACACCACGTTTTTGCATAGAGGGTCTGAGTCGCCGTCAATCGCGCCGTCTTAGCCATGTTAATCGTCATGACGTCGCGCGACTCAATTGCCACGTAACCTCCTACTTCGGGCGGCAGCGATTGCACATTGAAATGTTTGCGCTTCTGTATGCTCATAATATTCGGTTCCCATTATTGTTTGATCCATTAATTGTAGAAGATGCTGATAAAGCGGATTGGTCATTGAGCAGCGAGAATTGGGGCTATGGGAAACAACTGAGAAAAGCACCGTTAATCTCGTGTAGCTTGACGGAGTTGGATTTATCATTGTGGCATACTACCATTAAAATCGTTCTCCGGACTTGTTCTTGGCGTACTGAGCGCCGTAGGCGTCCAGTAGTAGGGAGTACGTACAAACGAATCCTAGATCCCGAGGAGGTCCTAAGCCTCAACTTCTGAGAAGGGGCATATTTACCTCTCGGCGCAAAAAAATTGCCGGTGGCAGACGCCATGCCCTGCATACGAGATCACGCCATCCACCTAACTCAGACCTCGGCCGAGGTAACTGGCGGGGGGCAAACCGGTGATAGGTCTCATTCTAGAGCACAACGTAATGATCACGTCTGGCGTAAGGGACATACACCGCGAGGCCGCCACACCGCGTCAACTCACTAAGAATAGGCTTATACCTAATTGCCTGACATTTTATGTTAGGAAGGGTACCTTTGGCTACAATCGATCAACGAGTGTGAGCAAGGCCTTTAAAGTCCGATCTGGACCCATTCACAAATCTGATCTGCGATTGCTCGAGTGACGGAGT	3.39E-06
SGE_ADFI	47	3	3017289-3110540	TTACCAATCTTAATTCAGAACCGGTAGTTTATATAGGGGGTAGACCGTATCCGCACGCTACGCTCGAAAGTAACTACAGAGTCATTTTTGCCTACAGTACGTTTTCTCTTTATAGGCAGTCTCTATT	3.46E-06
SGE_ADG	1834	1	275922211-276425999	CCAAGAGGAATCCCTTTTTTTTACGCGGACCCGAGGGGCCGTGCTCTACGGCGTAGTACTGGTAATTGGTGTTGCAAAACATCCGGCTAGGAAATGGTCGTTGAAGTATCTTAACGGGTATTCGGACTAGTGTACTGATG	1.86E-07
SGE_ADG	1832	1	274735024-275588282	TCCTTTCAAATAAGTACAGCGATGACAGCTCCTGCGCTTAGGCGGCAAAACGCGTGCGCTACATTGCACTGCAAACGGCTGGGTGGCACAAAGGAATATTGCTCATACTGGGGGCCACTCGACTCACGGTCGTACGAATAATCAATGCCCCATAGTGTGCCAGGTAACTTATGTCGCTCCGGCCAGTGCTTGCTGGAGTGCCGTGTCTGCCGTGAGCTGCAAATACGATCAAGCAGGTTACTTCGGGTGGATTCGGACAAGTGATGATATTCATACTTCGTGCCAACATTCACCGTGCCCCCGACCACCGCCCAGAATTACGAGAAAGACGAGAGGGTAATTTAAGAAACATCTCGGACAATTCAGTTCCTATTCCTTGGGCACCACCGAGTAGACGGCACTGTTTTTCGTCCTAGGTCTAGCAAGGAGTCCAAGGCATCAACTGACAAACTGATTCATCTGTGAGTGAAATATGATCCTGACGTTCTTGAGATGAACTCATGGG	2.54E-07
SGE_ADG	1193	10	61915547-61920091	GAAGCTTATACGGCAAATGTGGTGCGCTGG	2.91E-07
SGE_ADG	1467	4	17119823-17160785	GATTTGGCGGACAGCGTATCTCAGAAAGCCAGAAAATAGACACAAGTTACTGTCGTCGGCCAAAACAAGTGGGGACGGGTACAACAGGATGAGGGCAATCACCGTAACGACAGATAGTCTGCGATTCCGGAAAAGCCGTGCTGTTTCCTACTTCCAAAGATAGAACGTGCATAAATTTGGTCTCCT	9.12E-07

SGE_ADG	14	12	7980942-8179537	ATTTAAAACTCAGGTCACTGTCACCTAATCCCCACACAGCTCACTGCCCCGCTTGCTCCCCCCACCTACGTCCAAACCTGGGCTTGCGTTGTTATTGTTGTATTACCGGAAACAGCAGGGATTGGCAGCGCCGATTTGAAACACCGTGGGTACGTTGCTCGGGAGATGCACATTTCCCCTGCGCCGCTAGTTGCGGCACCTACACGTAGGCGCAGGGGCAAGTCGCCACTACCGTGCCAACGAGGCGACCATCTCGGGACCCGATAATTGTGCTTGTGTTCACTGCACGGGCCACCCGCCCCAAAATACAAGGGGGGCTTCGTGAACCCCCAGTGCAACCCTTCCACTCCCACAACCGCAGCGCGGCCGGGGGGCTTCTGCCAGCCGTCTGCAGCATCCAGGGATGTATGTCATTAGCTGAAGCGGGCCGACAGGATAGGTGTGCCTCACACTTCTGGGGGGCCCCAGGTGAGGGAATGCCGGCGGGCTGGACCA	1.44E-06
SGE_ADG	1698	9	20052831-20057732	TATAGTTTGCGTACATGTTCGCCCTTCCGCCTGGAGTTGC	2.07E-06
SGE_ADG	558	7	5811660-5837760	GGTACATCGCATGCACATGCTCCACGGATGCCCCAAGTCGT	2.15E-06
SGE_D100	2197	8	38588797-39104506	CACGCTGATTGGGGGTAGGATTAGTCTGACAAAAATTGGCGAGAGCCGAGACTCTTTGCCTTCAATCCATAGCTGCCAATCATCCAGTAAATTTTTCTATCTAACGAGCCTTAGTTCGCTCTTGTACCACGATATAACATCAGATGATACGGTCCGAGTAATTCTCACGCTGTGCGTCGGCGTTCACCTGTCACCGTGAAGGCTGTACCGATACCGATAACTCAACATTAGACCGTAAGGCCCGTGTCGAATAACTTTTATAACGCTCGCTCGTACATCGCGACTCCTGGGCGTTCAAGCCCATA	2.15E-07
SGE_D100	181	7	51827448-52540567	ACACCGACTACAAAACTCTCAGTAATTAACCAAAAGACATTCAGTAGGCCGAACGTCTAGGATAAGTTTCCTTAAATGCATCTCGCAATCGAATAGGCTCACGTTCGCGGGTTATGTACTTCGAATCACTTTACAGGTAGTAGGACACTAGAAGGAAGCTATTTCTTTCGTGACGCGACATTTCAACCCAGTCCAATCACCAGCAAGTAATCTTGAGTGACGACATCTGGGTCTCAACAGATACGACAATCTGCCCTTATTCCCTGCTTACGGATTGCATCCACCGGTCTCTCTGTCAACATATACGTATATAGTTGGGCTATCTAATTGAGGTACCATACATCTGGACGATATGGCCTTGGGGTCGCTACAAAACCACTAGGCGAACTGGGGGCATACAACCTAAGCTAAAAAATGACGCTCAGTAATAGACCTAATGCTAGAGGGACAGACAATGGATGCCGATTAGACATGTGGCTTTTCCTGCTTGGCGCGGTCTCCCATCGTACCGGCAAACCTTATTGAGTTGGGGAGCAGGGTTTCGTAAAGCCGAATCTAAAGAGAGTCACAATCGCTACACACCAAATACGTTGCCCACTCTCAAGGCACGTTATGGTGTTAGGAGCGACATCATCAGCATGCATGTGCAGGGCGCTTCAGGGTAGAGAGGCACTCCTCTGGGTGGTCGATTGGTGAACACTACTAAAGAACACATATATGACGGAATAACGAAGACTATCACGTGAAACGGGAAATTTAAGATGACTCGAACCATGGGGAAATAAGCAGCGGCTGTATCAGGAAGGCCAGTAATGCTCAATTGACCAGATGGGACAAGAACGCAAAGAAGACGCACAGAAAACTTATGAGGGCGCCGGGAACGGAGACAATAAGATAATCTTCATGATAAACGACCACAAGGTACAACGGAAGAGTAACATCAGACAACGGGTTCTTTCGGTACAACAGGGTTTTCTTTCAACTGAGCTCCCATGTGTGGGGAATAGAAGGCATACAACTGGCCGTGTTTAGCCGCACCAGTCCCCGCTTCCTATGCGCCCAACGCGCGAAGCGGACCTGCAGGTGTAGA	5.85E-07
SGE_D100	759	7	106237871-106507416	TTAGGCTAATATATCAGAAAGTTATAGGAGAGATTTTAGGAGGCCGACAATTGGAACGGCACCTAGAGAGCTATGTGTTTTATCCCACCAGAGCTTCGTAGCCATGTCGTAATAATATGGGCTGGGAGATCTAGTCCATTGCATAGGTTAGGAGGTTGAAC	6.67E-07
SGE_D100	1066	16	16761589-16929373	CTTGAAGTAATTCAGCGTTAGTCCAACTGGCCAGGCTCCAAGCACCAAGGCATGAATATTGTTATCCGATTCAATACGGTCCGGGCTACTCACGAATCGGGGATTCGCGTAAGTTATCCCGAGTACGGCCGGACTGCCACCAACGTTTAAGAATACACCATGGGAGAGCTATAGTTTCCTTCACTACCTCCCAGAACTCCGACCTTTTACTACAAGCGTGTCAGCTTTCCTGGCTCAGTGCTGCGTGAGCCCAGTTCCAGATGGAGGCCAAGACAGTGCGCATATGTGTTACCCAGCTATGTATCGTTTAACATGGTGCAGGTGCTTCCAAACTCTAGGAGGACCCCAACAATGTAGCGACGTTTTTGGCTACACCCGCATGTAGAACATCTTGCATATGAGCACCATCAGCGCCCCTGGGGTGCCATAGATCTACGCGCATGGGATTGGAGTAGTGACGAGAGCTTGCTAATATGCCACTGAAACAAACAGCAGCTGCATAAATACTTCCAGTTGCGCACGATCACTCTTTTTTCACAAAAGGAAAGAACGCAAGTAAACAACTAGTCACCCCAGCGGGCCATAGCTTGGGTGAGTACTAATTGAGAAAATTTTTTTCGGCACTTTGAACAAAACAATCCACTCATATTTCTTGATGTCTAAGTGCTTACGTGCTACGTGCTAC	1.24E-06

SGE_D100	1069	16	16941814-17012360	GGTACAAACGGCTTGCCAGGTGCCATCTCACCATCACTTTAGTCCGTGTATGATGGCTACGTTATCTAGGGGGCAGATCGGTCAGCTCAATAAATGGATGGCTATGCCGTTTTGTGTCTGATGGTCAATTATGACAGTAAAGCCCATCTCGGAATGCTGCTGAGTTATGGCTTCGGTCAGAGTT	1.24E-06
SGE_D100	1073	16	17014404-17022242	AGACTGCTAAACGGGAGTACCCTCACGTATATCCCTTATCTTCAAAGAGAAGAGCTCTTAACTTACACAAACCGATGTT	1.24E-06
SGE_D100	1073	16	17014404-17022242	AGCCTGCTAAAAAGAATTTCTCCTATGAGTGCGTTGCCGTGCTCTGGGGAAAAGCACCTAGTCAATGTGGGTTTGGACC	1.24E-06
SGE_D100	1078	16	17024403-17033863	AAGGCTTGTTTAACGGGATAAGCCAAGTCAGCGTTTGTCCATCGGCCTAATATATA	1.24E-06
SGE_D100	1078	16	17024403-17033863	AAGGCTTGTTCGGGAATACGGAGTGCCGAATTCCCCACCGGAGAGTTCGGAATAAG	1.24E-06
SGE_D100	1083	16	17035724-17047602	AGAGACTGAGCGTAGTCTTGGCGGCGTGGGAA	1.24E-06
SGE_D100	1085	16	17048802-17059077	TGAAATGTCCACCTACGTTATATCATCTATCATCCA	1.24E-06
SGE_D100	1086	16	17059099-17075409	TAACGTGCTCTCTGATATGAGATTATCAGCTCAGTTTCAAGTATTCCCAGATGTCGCTAT	1.24E-06
SGE_D100	1086	16	17059099-17075409	CAGTCTTTTCCCTGAAAAGACACTATCAGCTCAGTTTCAAGTACTCCTAGATGTCTTTAG	1.24E-06
SGE_D100	1087	16	17075616-17093253	ACTCATGCACCAAGGTATGTCGGTCCCGCAGGAAATTCTAACCCGTATTGCTACTGTGTGCCGTGTTGCGCACTCTCAAGACCTCCAATAACCCTTCACGTACGTTGGCGCCGGTATAGGTTG	1.24E-06
SGE_D100	1095	16	17106392-17116817	ATTGTAATTCAGACCATGTCTGGTATGCTTGGAGTGAAAGGTTGTTACTCTGTATGAACGGATTGTAGTT	1.24E-06
SGE_D100	1358	9	13517556-13518554	CGCTACTCGCTT	1.52E-06
SGE_RFI	931	4	132774639-132786416	CCAACAGGGGCAGAAGTGTTGTCATAGTCGGTGCTCTTTTAG	3.31E-06
SGE_FS	2691	1	21541710-21543053	GACTCGCAACATA	2.33E-07
SGE_FS	2692	1	21543085-21565658	CTGTTCAACCTTGCATTCGCGCTGTTAGA	2.33E-07
SGE_FS	1220	1	209040701-209088366	CACACATTCCCAGGAAGATTCCGCATTTATCATATGTGAATTATCAGAAGCTCATGGCCGACGATTAATGCTAGATAAGTTCTGAAGCTGACGATTGAGAATCTAAAGCGGGACTCAGTACTCTCT	7.80E-07
SGE_TPD	67	1	36744464-36747949	CTTTTAGTGTCCTAAAAAGCCCTCAGACCAAACA	1.47E-06
SGE_TPD	1270	5	15864572-15959895	TAGGCCGGTCACGGGAGCGTCGCGAAGTTCGCGCTCTAAGCAAATCTCCCCAGCCTGAGGCCTTACGCATTGCGAAATCTAGGGTAGAAGGAGTCAGATCCCAAAATAATTAGACCAAAACGAGTCCTTGATATCATCGCCCCCTGGACGCGACGAAGGCGACCCTATTTGTCCAGAGCTCGTTGTTGCCTGCAACATGACTGTTTATGAATACAGACTCCGGATTTTCATGTCTGCGTTAGTTATTTTCCTGCGTTTCGTGATCATGAAAGCTTATTGCGTGCAGTGCGGCCCGTCCTATGCTTATGTCTTCTGCGAACGGAAACTTAAACCTAAACATCGAAGTTTAAGTTAATTCATACGTGGAAGCTACCAGGGAACAATGGCAACTCACGCATAAACCACGGGCGAGTTCATCCGCTCCGGGCGTCTCATCATGCAGTAATAAGAATTCAGCATACACAATTCATGAAAGATTGAAGCCAACATCATGAAAGTTGGGTCGCTCTCGCGGATTCGG	1.78E-06
SGE_TPD	580	10	24658634-24668970	TAGTAAACTTTGAAGAGGAAAGGCGCGCCAAGAGTAAA	1.62E-06

Supplementary Table 5. Comparative mapping of significant chromosome region with previous QTLs reported in the pig QTL database (as of July 1,

Identified significant chromosome regions	Trait	Related QTL ID	QTL location range(bp)	Details
Chr6: 45.95–46.95 Mb; Chr6: 25.15–26.15 Mb; Chr6: 18.14–19.14 Mb	Alkaline phosphatase activity	QTL:15097	Chr.6:17750243-152260387	Trait:Health>>blood parameters>>Alkaline phosphatase activity|CMO:serum alkaline phosphatase activity level
Chr6: 18.14–19.14 Mb	Mean corpuscular hemoglobin content	QTL:5425	Chr.6:2352681-19536155	Trait:Health>>blood parameters>>Mean corpuscular hemoglobin content|CMO:mean corpuscular hemoglobin level
Chr6: 18.14–19.14 Mb	Time spent socializing	QTL:5926	Chr.6:2352681-19536155	Trait:Exterior>>Behavioral>>Time spent socializing|VT:social interaction
Chr6: 18.14–19.14 Mb	CO2 partial pressure	QTL:6329	Chr.6:2352681-19536155	Trait:Health>>blood parameters>>CO2 partial pressure|VT:blood gas amount|CMO:partial pressure of blood carbon dioxide (Pco2)
Chr6: 18.14–19.14 Mb	PRRSV susceptibility	QTL:31808	Chr.6:18019907-18982722	Trait:Health>>Disease susceptibility>>PRRSV susceptibility|CMO:disease process measurement
Chr6: 18.14–19.14 Mb	Body weight	QTL:65070	Chr.6:17461058-19679106	Trait:Production>>Growth>>Body weight|VT:body mass|CMO:body
Chr6: 18.14–19.14 Mb	Average daily gain	QTL:28891	Chr.6:18653832-18653872	Trait:Production>>Growth>>Average daily gain|VT:postnatal growth trait|CMO:average daily body weight gain
Chr6: 45.95–46.95 Mb; Chr6: 25.15–26.15 Mb; Chr6: 18.14–19.14 Mb	Body weight (end of test)	QTL:5201	Chr.6:9242013-138477175	Trait:Production>>Growth>>Body weight (end of test)|VT:body mass|CMO:body weight
Chr6: 45.95–46.95 Mb; Chr6: 25.15–26.15 Mb; Chr6: 18.14–19.14 Mb	Actinobacillus pleuropneumoniae susceptibility	QTL:37574	Chr.6:9303695-83952943	Trait:Health>>Disease susceptibility>>Actinobacillus pleuropneumoniae susceptibility|CMO:disease process measurement
Chr6: 45.95–46.95 Mb; Chr6: 25.15–26.15 Mb; Chr6: 18.14–19.14 Mb	Actinobacillus pleuropneumoniae susceptibility	QTL:37566	Chr.6:9132652-83952943	Trait:Health>>Disease susceptibility>>Actinobacillus pleuropneumoniae susceptibility|CMO:disease process measurement
Chr6: 18.14–19.14 Mb	Average daily gain	QTL:332	Chr.6:8442460-19536155	Trait:Production>>Growth>>Average daily gain|VT:postnatal growth trait|CMO:average daily body weight gain
Chr6: 18.14–19.14 Mb	Average daily gain	QTL:343	Chr.6:8442460-19536155	Trait:Production>>Growth>>Average daily gain|VT:postnatal growth trait|CMO:average daily body weight gain
Chr6: 18.14–19.14 Mb	Pseudorabies susceptibility	QTL:304	Chr.6:9872228-19536155	Trait:Health>>Disease susceptibility>>Pseudorabies susceptibility|CMO:disease process measurement
Chr6: 18.14–19.14 Mb	Time in feeder per day	QTL:3893	Chr.6:18536951-18686071	Trait:Production>>feed intake>>Time in feeder per day|CMO:food intake duration
Chr6: 45.95–46.95 Mb; Chr6: 25.15–26.15 Mb; Chr6: 18.14–19.14 Mb	Osteochondrosis score	QTL:17641	Chr.6:11886408-74405304	Trait:Exterior>>defects>>Osteochondrosis score|VT:joint morphology trait|CMO:disease process measurement
Chr6: 45.95–46.95 Mb; Chr6: 25.15–26.15 Mb; Chr6: 18.14–19.14 Mb	Body weight (46 days)	QTL:21240	Chr.6:8247419-80284968	Trait:Production>>Growth>>Body weight (46 days)|VT:body mass|CMO:body weight
Chr6: 25.15–26.15 Mb; Chr6: 18.14–19.14 Mb	Average daily gain	QTL:5699	Chr.6:2352681-29979924	Trait:Production>>Growth>>Average daily gain|VT:postnatal growth trait|CMO:average daily body weight gain
Chr6: 45.95–46.95 Mb; Chr6: 25.15–26.15 Mb; Chr6: 18.14–19.14 Mb	Salmonella count in spleen	QTL:11562	Chr.6:9957192-129740986	Trait:Health>>Pathogens and parasites>>Salmonella count in spleen|VT:response to bacterial infection trait
Chr6: 45.95–46.95 Mb; Chr6: 25.15–26.15 Mb; Chr6: 18.14–19.14 Mb	Hind leg conformation	QTL:17640	Chr.6:11856794-72850194	Trait:Exterior>>conformation>>Hind leg conformation|VT:hindlimb conformation trait
Chr6: 45.95–46.95 Mb; Chr6: 25.15–26.15 Mb; Chr6: 18.14–19.14 Mb	Lactate dehydrogenase level	QTL:15099	Chr.6:18610219-123963269	Trait:Health>>blood parameters>>Lactate dehydrogenase level|VT:blood lactate dehydrogenase amount|CMO:blood lactate dehydrogenase activity level
Chr6: 45.95–46.95 Mb; Chr6: 25.15–26.15 Mb	Average daily gain	QTL:186	Chr.6:23594516-89095314	Trait:Production>>Growth>>Average daily gain|VT:postnatal growth trait|CMO:average daily body weight gain
Chr6: 25.15–26.15 Mb	Body weight	QTL:65072	Chr.6:24198500-26137432	Trait:Production>>Growth>>Body weight|VT:body mass|CMO:body
Chr6: 45.95–46.95 Mb; Chr6: 25.15–26.15 Mb	Average daily gain	QTL:3937	Chr.6:20981250-85846150	Trait:Production>>Growth>>Average daily gain|VT:postnatal growth trait|CMO:average daily body weight gain
Chr6: 45.95–46.95 Mb	Mean corpuscular hemoglobin concentration	QTL:27359	Chr.6:46003068-46003108	Trait:Health>>blood parameters>>Mean corpuscular hemoglobin concentration|CMO:mean corpuscular hemoglobin concentration

Chr6: 45.95–46.95 Mb	Mean platelet volume	QTL:37813	Chr.6:46003068-46003108	Trait:Health>>blood parameters>>Mean platelet volume|VT:platelet size trait|CMO:mean platelet volume
Chr6: 45.95–46.95 Mb	Mean corpuscular hemoglobin concentration	QTL:27347	Chr.6:46250497-46250537	Trait:Health>>blood parameters>>Mean corpuscular hemoglobin concentration|CMO:mean corpuscular hemoglobin concentration
Chr6: 45.95–46.95 Mb	Mean corpuscular hemoglobin concentration	QTL:27360	Chr.6:46270939-46270979	Trait:Health>>blood parameters>>Mean corpuscular hemoglobin concentration|CMO:mean corpuscular hemoglobin concentration
Chr6: 45.95–46.95 Mb	Mean platelet volume	QTL:37817	Chr.6:46270939-46270979	Trait:Health>>blood parameters>>Mean platelet volume|VT:platelet size trait|CMO:mean platelet volume
Chr6: 45.95–46.95 Mb	Mean platelet volume	QTL:37841	Chr.6:46687751-46687791	Trait:Health>>blood parameters>>Mean platelet volume|VT:platelet size trait|CMO:mean platelet volume
Chr6: 45.95–46.95 Mb	Mean platelet volume	QTL:37799	Chr.6:46893572-46893612	Trait:Health>>blood parameters>>Mean platelet volume|VT:platelet size trait|CMO:mean platelet volume
Chr6: 45.95–46.95 Mb	Average daily gain	QTL:16850	Chr.6:29979924-47267662	Trait:Production>>Growth>>Average daily gain|VT:postnatal growth trait|CMO:average daily body weight gain
Chr6: 45.95–46.95 Mb	Creatine kinase level	QTL:1045	Chr.6:29979924-86655461	Trait:Health>>blood parameters>>Creatine kinase level|VT:blood creatine kinase amount|CMO:blood creatine kinase activity level
Chr6: 45.95–46.95 Mb	CD4-positive leukocyte percentage	QTL:17841	Chr.6:35868354-52757738	Trait:Health>>Immune capacity>>CD4-positive leukocyte percentage|VT:leukocyte quantity
Chr6: 45.95–46.95 Mb	CD4-positive/CD8-positive leukocyte ratio	QTL:17842	Chr.6:35868354-52757738	Trait:Health>>Immune capacity>>CD4-positive/CD8-positive leukocyte ratio|VT:leukocyte quantity
Chr6: 45.95–46.95 Mb	Creatine kinase level	QTL:1041	Chr.6:45448178-77813565	Trait:Health>>blood parameters>>Creatine kinase level|VT:blood creatine kinase amount|CMO:blood creatine kinase activity level
Chr6: 45.95–46.95 Mb	Mean corpuscular volume	QTL:7223	Chr.6:46571301-47119297	Trait:Health>>blood parameters>>Mean corpuscular volume|VT:erythrocyte size trait|CMO:mean corpuscular volume
Chr6: 45.95–46.95 Mb	Time spent feeding	QTL:5911	Chr.6:34464154-129740986	Trait:Exterior>>Behavioral>>Time spent feeding|VT:eating behavior trait
Chr6: 45.95–46.95 Mb	Alkaline phosphatase activity	QTL:6347	Chr.6:34464154-129740986	Trait:Health>>blood parameters>>Alkaline phosphatase activity|CMO:serum alkaline phosphatase activity level
Chr6: 45.95–46.95 Mb	Calcium level	QTL:6376	Chr.6:34464154-129740986	Trait:Health>>blood parameters>>Calcium level|VT:blood calcium amount|CMO:blood calcium level
Chr6: 45.95–46.95 Mb	Average daily gain	QTL:185	Chr.6:30938918-88161912	Trait:Production>>Growth>>Average daily gain|VT:postnatal growth trait|CMO:average daily body weight gain
Chr6: 45.95–46.95 Mb	Platelet count	QTL:7499	Chr.6:34464154-129740986	Trait:Health>>blood parameters>>Platelet count|VT:platelet quantity|CMO:platelet count
Chr6: 45.95–46.95 Mb	Platelet count	QTL:7500	Chr.6:34464154-129740986	Trait:Health>>blood parameters>>Platelet count|VT:platelet quantity|CMO:platelet count
Chr8: 79.01–80.01Mb	Melanoma susceptibility	QTL:7579	Chr.8:10844129-148491826	Trait:Health>>Disease susceptibility>>Melanoma susceptibility|CMO:disease process measurement
Chr8: 79.01–80.01Mb	Body weight (34 weeks)	QTL:21254	Chr.8:7495097-92309019	Trait:Production>>Growth>>Body weight (34 weeks)|VT:body mass|CMO:body weight
Chr8: 79.01–80.01Mb	Body weight (30 weeks)	QTL:21253	Chr.8:4904068-124426079	Trait:Production>>Growth>>Body weight (30 weeks)|VT:body mass|CMO:body weight
Chr8: 79.01–80.01Mb	Mean corpuscular hemoglobin concentration	QTL:22138	Chr.8:31525187-79808773	Trait:Health>>blood parameters>>Mean corpuscular hemoglobin concentration|CMO:mean corpuscular hemoglobin concentration
Chr8: 79.01–80.01Mb	Mean corpuscular volume	QTL:22144	Chr.8:34394607-79128136	Trait:Health>>blood parameters>>Mean corpuscular volume|VT:erythrocyte size trait|CMO:mean corpuscular volume
Chr8: 79.01–80.01Mb	Mean corpuscular hemoglobin concentration	QTL:22162	Chr.8:34904211-79187782	Trait:Health>>blood parameters>>Mean corpuscular hemoglobin concentration|CMO:mean corpuscular hemoglobin concentration
Chr8: 79.01–80.01Mb	Body weight (birth)	QTL:29699	Chr.8:26534249-90653103	Trait:Production>>Growth>>Body weight (birth)|VT:body mass|CMO:body weight
Chr8: 79.01–80.01Mb	Mean corpuscular volume	QTL:22164	Chr.8:34394607-84490713	Trait:Health>>blood parameters>>Mean corpuscular volume|VT:erythrocyte size trait|CMO:mean corpuscular volume

Chr8: 79.01–80.01Mb	Body weight (3 weeks)	QTL:29700	Chr.8:11308413-108610930	Trait:Production>>Growth>>Body weight (3 weeks)|VT:body mass|CMO:body weight
Chr8: 79.01–80.01Mb	Red blood cell count	QTL:15053	Chr.8:39840424-90653103	Trait:Health>>blood parameters>>Red blood cell count|VT:erythrocyte quantity|CMO:red blood cell count
Chr8: 79.01–80.01Mb	Mean corpuscular hemoglobin content	QTL:6546	Chr.8:79969240-80490417	Trait:Health>>blood parameters>>Mean corpuscular hemoglobin content|CMO:mean corpuscular hemoglobin level
Chr8: 79.01–80.01Mb	Mean corpuscular hemoglobin content	QTL:6547	Chr.8:79969240-80490417	Trait:Health>>blood parameters>>Mean corpuscular hemoglobin content|CMO:mean corpuscular hemoglobin level
Chr8: 79.01–80.01Mb	Mean corpuscular hemoglobin content	QTL:6548	Chr.8:79969240-80490417	Trait:Health>>blood parameters>>Mean corpuscular hemoglobin content|CMO:mean corpuscular hemoglobin level
Chr8: 79.01–80.01Mb	Mean corpuscular volume	QTL:6551	Chr.8:79969240-80490417	Trait:Health>>blood parameters>>Mean corpuscular volume|VT:erythrocyte size trait|CMO:mean corpuscular volume
Chr8: 79.01–80.01Mb	CD4-positive leukocyte percentage	QTL:17844	Chr.8:78781463-90653103	Trait:Health>>Immune capacity>>CD4-positive leukocyte percentage|VT:leukocyte quantity
Chr8: 79.01–80.01Mb	CD4-positive/CD8-positive leukocyte ratio	QTL:17845	Chr.8:78781463-90653103	Trait:Health>>Immune capacity>>CD4-positive/CD8-positive leukocyte ratio|VT:leukocyte quantity
Chr8: 79.01–80.01Mb	Platelet distribution width	QTL:22169	Chr.8:66027033-79128136	Trait:Health>>blood parameters>>Platelet distribution width|VT:platelet size trait|CMO:platelet distribution width
Chr8: 79.01–80.01Mb	Mean corpuscular volume	QTL:22142	Chr.8:66027033-79512659	Trait:Health>>blood parameters>>Mean corpuscular volume|VT:erythrocyte size trait|CMO:mean corpuscular volume
Chr8: 79.01–80.01Mb	Mean corpuscular volume	QTL:22143	Chr.8:66027033-79512659	Trait:Health>>blood parameters>>Mean corpuscular volume|VT:erythrocyte size trait|CMO:mean corpuscular volume
Chr8: 79.01–80.01Mb	Mean corpuscular volume	QTL:22141	Chr.8:66027033-79512659	Trait:Health>>blood parameters>>Mean corpuscular volume|VT:erythrocyte size trait|CMO:mean corpuscular volume
Chr8: 79.01–80.01Mb	Hemoglobin	QTL:4277	Chr.8:39840424-108610930	Trait:Health>>blood parameters>>Hemoglobin|VT:blood hemoglobin amount|CMO:hemoglobin concentration
Chr8: 79.01–80.01Mb	Red blood cell count	QTL:22146	Chr.8:66027033-84490713	Trait:Health>>blood parameters>>Red blood cell count|VT:erythrocyte quantity|CMO:red blood cell count
Chr8: 79.01–80.01Mb	Red blood cell count	QTL:22166	Chr.8:66027033-85118335	Trait:Health>>blood parameters>>Red blood cell count|VT:erythrocyte quantity|CMO:red blood cell count
Chr8: 79.01–80.01Mb	Alkaline phosphatase activity	QTL:15105	Chr.8:11308413-124156612	Trait:Health>>blood parameters>>Alkaline phosphatase activity|CMO:serum alkaline phosphatase activity level
Chr8: 79.01–80.01Mb	Red blood cell count	QTL:29655	Chr.8:79682665-79682705	Trait:Health>>blood parameters>>Red blood cell count|VT:erythrocyte quantity|CMO:red blood cell count
Chr8: 79.01–80.01Mb	Mean corpuscular hemoglobin content	QTL:29656	Chr.8:79682665-79682705	Trait:Health>>blood parameters>>Mean corpuscular hemoglobin content|CMO:mean corpuscular hemoglobin level
Chr8: 79.01–80.01Mb	Mean corpuscular volume	QTL:29657	Chr.8:79682665-79682705	Trait:Health>>blood parameters>>Mean corpuscular volume|VT:erythrocyte size trait|CMO:mean corpuscular volume
Chr8: 79.01–80.01Mb	Red blood cell count	QTL:29658	Chr.8:79687998-79688038	Trait:Health>>blood parameters>>Red blood cell count|VT:erythrocyte quantity|CMO:red blood cell count
Chr8: 79.01–80.01Mb	Mean corpuscular volume	QTL:29662	Chr.8:79687998-79688038	Trait:Health>>blood parameters>>Mean corpuscular volume|VT:erythrocyte size trait|CMO:mean corpuscular volume
Chr8: 79.01–80.01Mb	Bilirubin level	QTL:6374	Chr.8:46427317-120532322	Trait:Health>>blood parameters>>Bilirubin level|VT:blood bilirubin amount|CMO:blood bilirubin level
Chr8: 79.01–80.01Mb	Days to 100 kg	QTL:62291	Chr.8:67430450-107954353	Trait:Production>>Growth>>Days to 100 kg|VT:postnatal growth trait
Chr8: 79.01–80.01Mb	Days to 100 kg	QTL:62292	Chr.8:67546715-107954353	Trait:Production>>Growth>>Days to 100 kg|VT:postnatal growth trait
Chr8: 79.01–80.01Mb	Days to 100 kg	QTL:62293	Chr.8:68054982-107954353	Trait:Production>>Growth>>Days to 100 kg|VT:postnatal growth trait
Chr8: 79.01–80.01Mb	Days to 100 kg	QTL:62294	Chr.8:68093977-107954353	Trait:Production>>Growth>>Days to 100 kg|VT:postnatal growth trait
Chr8: 79.01–80.01Mb	Glucose level	QTL:15106	Chr.8:39840424-124156612	Trait:Health>>blood parameters>>Glucose level|VT:blood glucose amount|CMO:blood glucose level

<table>
<tr><td>Chr8: 79.01–80.01Mb</td><td>Toll-like receptor 9 level</td><td>QTL:17620</td><td>Chr.8:77429212-124156612</td><td>Trait:Health>>Immune capacity>>Toll-like receptor 9 level|VT:toll-like receptor 9 protein amount</td></tr>
</table>

Supplementary Table 6. The identified significant GO terms of candidate genes for socially affected traits in pigs

Category	Go Term	Gene Count	Fold Enrichment	Genes	P-value
GOTERM_BP_DIRECT	GO:0006355	5	7.24	ZNF527, ZNF569, ZNF146, NEUROG1, ZNF461	0.003676
GOTERM_BP_DIRECT	GO:0007156	3	22.56	CDH1, CDH3, CDH11	0.006982
GOTERM_CC_DIRECT	GO:0005622	4	4.17	ZNF527, ZNF567, ZNF569, ZNF461	0.060597
GOTERM_MF_DIRECT	GO:0003676	5	6.68	ZNF527, POLR2I, ZNF569, ZNF146, ZNF461	0.004673
GOTERM_MF_DIRECT	GO:0046872	6	4.44	ZNF527, MT1A, ZNF567, ZNF569, ZNF146, ZNF461	0.007030
GOTERM_MF_DIRECT	GO:0005509	4	4.93	CAPNS1, CDH1, CDH3, CDH11	0.039216
