# Supplementary material for: A combined GWAS approach reveals key loci for socially-affected traits in Yorkshire pigs
Source: Commun Biol. 2021 Jul 20;4:891. doi: 10.1038/s42003-021-02416-3 (PMC8292486; doi:10.1038/s42003-021-02416-3)
Supplement: Supplementary file 4 — Description of Additional Supplementary Files [file 42003_2021_2416_MOESM4_ESM.pdf]

**Description of Additional Supplementary Files**

**File name:** Supplementary data 1-2

**Description:**

Supplementary data 1. Summary and mapping statistics of a data set including whole-genome sequences from 40 Yorkshire pigs and 20 Landrace pigs.

Supplementary Data 2. Single-locus GWAS results for DGE and SGE using imputed WGS data (3.25E-07< P < 1.63E-08).
