# Supplementary material for: A combined GWAS approach reveals key loci for socially-affected traits in Yorkshire pigs
Source: Commun Biol. 2021 Jul 20;4:891. doi: 10.1038/s42003-021-02416-3 (PMC8292486; doi:10.1038/s42003-021-02416-3)
Supplement: Supplementary file 5 — Reporting Summary [file 42003_2021_2416_MOESM5_ESM.pdf]

nature research

Corresponding author(s): Guoqing Tang

Last updated by author(s): Jan 21, 2021

Reporting Summary

Nature Research wishes to improve the reproducibility of the work that we publish. This form provides structure for consistency and transparency in reporting. For further information on Nature Research policies, see our Editorial Policies and the Editorial Policy Checklist.

Statistics

For all statistical analyses, confirm that the following items are present in the figure legend, table legend, main text, or Methods section.

n/a	Confirmed	
[ ]	[x]	The exact sample size (*n*) for each experimental group/condition, given as a discrete number and unit of measurement
[ ]	[x]	A statement on whether measurements were taken from distinct samples or whether the same sample was measured repeatedly
[ ]	[x]	The statistical test(s) used AND whether they are one- or two-sided<br>*Only common tests should be described solely by name; describe more complex techniques in the Methods section.*
[x]	[ ]	A description of all covariates tested
[x]	[ ]	A description of any assumptions or corrections, such as tests of normality and adjustment for multiple comparisons
[ ]	[x]	A full description of the statistical parameters including central tendency (e.g. means) or other basic estimates (e.g. regression coefficient) AND variation (e.g. standard deviation) or associated estimates of uncertainty (e.g. confidence intervals)
[ ]	[x]	For null hypothesis testing, the test statistic (e.g. *F*, *t*, *r*) with confidence intervals, effect sizes, degrees of freedom and *P* value noted<br>*Give P values as exact values whenever suitable.*
[x]	[ ]	For Bayesian analysis, information on the choice of priors and Markov chain Monte Carlo settings
[x]	[ ]	For hierarchical and complex designs, identification of the appropriate level for tests and full reporting of outcomes
[x]	[ ]	Estimates of effect sizes (e.g. Cohen's *d*, Pearson's *r*), indicating how they were calculated

Our web collection on statistics for biologists contains articles on many of the points above.

Software and code

Policy information about availability of computer code

Data collection	GATK software v3.5; FastQC v0.11.9;BurrowsWheeler Aligner software (BWA, version 0.7.15);
Data analysis	DMU software; PLINK software v1.90; Beagle software v5.1; SAMtools v1.19; GEMMA software; R software v4.0.2

For manuscripts utilizing custom algorithms or software that are central to the research but not yet described in published literature, software must be made available to editors and reviewers. We strongly encourage code deposition in a community repository (e.g. GitHub). See the Nature Research guidelines for submitting code & software for further information.

Data

Policy information about availability of data

All manuscripts must include a data availability statement. This statement should provide the following information, where applicable:

- Accession codes, unique identifiers, or web links for publicly available datasets
- A list of figures that have associated raw data
- A description of any restrictions on data availability

The datasets supporting the results of this article are included within the article. All genotypic, phenotype data and supplemental material were deposited in https://figshare.com/s/23843254cfba1097cabd

Field-specific reporting

Life sciences study design

All studies must disclose on these points even when the disclosure is negative.

Sample size	No statistical tests were used to predetermine sample size. Sample sizes were similar to those commonly used in this research field.
Data exclusions	For Illumina Porcine 50K SNP Chip, SNPs with call rate< 0.90, MAF< 0.05, HWE<10–6, and the SNPs with no position information and located on sex autosomes and were excluded. For WGS data, SNPs with MAF < 0.05, missing rate >0.1, HWE > 10–6, read depth (dp) < 6 and the SNPs located on sex autosomes were excluded. For genotype imputation, SNPs with a Beagle R2 < 0.8 were excluded. For haplotype loci, haplotype alleles with MAF < 0.01 were excluded.
Replication	The datasets supporting the results of this article are included within the article. All genotypic, phenotype data and supplemental material were deposited at the figshare repository (https://doi.org/10.6084/m9.figshare.12611786). All other data are available from the corresponding authors upon reasonable request.
Randomization	Randomization was performed
Blinding	Blinding was not performed to ensure correct assignment of the data to the measured constructs and/or experimental conditions.

Reporting for specific materials, systems and methods

We require information from authors about some types of materials, experimental systems and methods used in many studies. Here, indicate whether each material, system or method listed is relevant to your study. If you are not sure if a list item applies to your research, read the appropriate section before selecting a response.

Materials & experimental systems

n/a	Involved in the study	
☒	☐	Antibodies
☒	☐	Eukaryotic cell lines
☒	☐	Palaeontology and archaeology
☐	☒	Animals and other organisms
☒	☐	Human research participants
☒	☐	Clinical data
☒	☐	Dual use research of concern

Methods

n/a	Involved in the study	
☒	☐	ChIP-seq
☒	☐	Flow cytometry
☒	☐	MRI-based neuroimaging

Animals and other organisms

Policy information about studies involving animals; ARRIVE guidelines recommended for reporting animal research

Laboratory animals	All animals were from a commercial pig performance testing station of New Hope Group, Co., Ltd. (Sichuan, China).
Wild animals	The study did not involve wild animals.
Field-collected samples	The study did not involve samples collected from the field.
Ethics oversight	All experimental procedures were performed in accordance with the Institutional Review Board (IRB14044) and the Institutional Animal Care and Use Committee of the Sichuan Agricultural University under permit number DKY-B20140302.

Note that full information on the approval of the study protocol must also be provided in the manuscript.
